# Diversity across major and candidate genes in European local pig breeds

**DOI:** 10.1371/journal.pone.0207475

**Published:** 2018-11-20

**Authors:** María Muñoz, Riccardo Bozzi, Fabián García, Yolanda Núñez, Claudia Geraci, Alessandro Crovetti, Juan García-Casco, Estefania Alves, Martin Škrlep, Rui Charneca, Jose M. Martins, Raquel Quintanilla, Joan Tibau, Goran Kušec, Ivona Djurkin-Kušec, Marie J. Mercat, Juliette Riquet, Jordi Estellé, Christoph Zimmer, Violeta Razmaite, Jose P. Araujo, Čedomir Radović, Radomir Savić, Danijel Karolyi, Maurizio Gallo, Marjeta Čandek-Potokar, Luca Fontanesi, Ana I. Fernández, Cristina Óvilo

**Affiliations:** 1 Departamento Mejora Genética Animal, INIA, Madrid, Spain; 2 Università degli Studi di Firenze, Firenze, Italy; 3 Department of Agricultural and Food Sciences, University of Bologna, Bologna, Italy; 4 Kmetijski inštitut Slovenije, Ljubljana, Slovenia; 5 Instituto de Ciências Agrárias e Ambientais Mediterrânicas (ICAAM), Universidade de Évora, Évora, Portugal; 6 Programa de Genética y Mejora Animal, IRTA, Barcelona, Spain; 7 University of Osijek, Faculty of Agrobiotechnical Sciences, Osijek, Croatia; 8 Institut du Porc, IFIP, Le Rheu, France; 9 Génétique Physiologie et Système d’Elevage, INRA, Castanet-Tolosan, France; 10 GABI, INRA, AgroParisTech, Université Paris-Saclay, Jouy-en-Josas, France; 11 Bäuerliche Erzeugergemeinschaft Schwäbisch Hall, Schwäbisch Hall, Germany; 12 Animal Science Institute, Lithuanian University of Health Sciences, Baisogala, Lithuania; 13 Instituto Politecnico de Viana do Castelo, Viana do Castelo, Portugal; 14 Institute for Animal Husbandry-Pig Research Department, Belgrade-Zemun, Serbia; 15 University of Belgrade, Faculty of agriculture, Belgrade-Zemun, Serbia; 16 Department of animal science, Faculty of agriculture, University of Zagreb, Zagreb, Croatia; 17 Associazione Nazionale Allevatori Suini (ANAS), Roma, Italy; University of Illinois, UNITED STATES

## Abstract

The aim of this work was to analyse the distribution of causal and candidate mutations associated to relevant productive traits in twenty local European pig breeds. Also, the potential of the SNP panel employed for elucidating the genetic structure and relationships among breeds was evaluated. Most relevant genes and mutations associated with pig morphological, productive, meat quality, reproductive and disease resistance traits were prioritized and analyzed in a maximum of 47 blood samples from each of the breeds (Alentejana, Apulo-Calabrese, Basque, Bísara, Majorcan Black, Black Slavonian (Crna slavonska), Casertana, Cinta Senese, Gascon, Iberian, Krškopolje (Krškopoljski), Lithuanian indigenous wattle, Lithuanian White Old Type, Mora Romagnola, Moravka, Nero Siciliano, Sarda, Schwäbisch-Hällisches Schwein (Swabian Hall pig), Swallow-Bellied Mangalitsa and Turopolje). We successfully analyzed allelic variation in 39 polymorphisms, located in 33 candidate genes. Results provide relevant information regarding genetic diversity and segregation of SNPs associated to production and quality traits. Coat color and morphological trait-genes that show low level of segregation, and fixed SNPs may be useful for traceability. On the other hand, we detected SNPs which may be useful for association studies as well as breeding programs. For instance, we observed predominance of alleles that might be unfavorable for disease resistance and boar taint in most breeds and segregation of many alleles involved in meat quality, fatness and growth traits. Overall, these findings provide a detailed catalogue of segregating candidate SNPs in 20 European local pig breeds that may be useful for traceability purposes, for association studies and for breeding schemes. Population genetic analyses based on these candidate genes are able to uncover some clues regarding the hidden genetic substructure of these populations, as the extreme genetic closeness between Iberian and Alentejana breeds and an uneven admixture of the breeds studied. The results are in agreement with available knowledge regarding breed history and management, although largest panels of neutral markers should be employed to get a deeper understanding of the population’s structure and relationships.

## Introduction

Modern pig production is mostly based on highly-selected genotypes from lean breeds, farmed in intensive production systems for provisioning the market with fresh pork. On the other hand, the use of local examples of pig breeds is usually associated with local forms of pig husbandry and processing of their meat into high-quality products, often dry-cured. The most representative local pig breed is the Iberian raised in the southwest of the Iberian Peninsula, but there are many other breeds reared in European countries. These breeds are in general characterized by a good adaptation to specific environments, high potential for fat deposition and characteristic meat quality, mostly related to high intramuscular or intermuscular fat content, which are associated with high quality pork productions.

Conservation of animal genetic resources depends on current and future economic relevance of autochthonous breeds but also on scientific importance, the cultural and historical value, adaptation to environment and contribution to the development of local communities and marginal areas. Local breeds, although showing economic and social relevance, may be threatened by inefficient utilization [1] and the development of sustainable pork chains is a main goal. In order to help breed promotion and sustainability, a deep phenotypic and genetic characterization is needed.

Neutral markers like microsatellites have been recommended by FAO for genetic characterization of animal breeds [1]. However, the relationship between variability at neutral marker loci and productive or adaptive traits is unclear [2]. It is also known that in domestic animals some important phenotypic differences between breeds such as coat color, vertebrae number and production traits, may be due to differences at few loci [3]. Hence, sequence polymorphisms within major or candidate genes may be more useful for the purposes of genetic characterization, breed differentiation and development of new breeding procedures aiming to manage genetic information.

In this study, we have investigated, in 20 local breeds, allele frequency distribution of causative polymorphisms and polymorphisms associated with relevant traits in a total of 33 genes selected from previous studies carried out in cosmopolitan breeds. The analysis was focused on variants in candidate genes known to affect the regulation of growth and appetite, meat quality, coat color, reproduction and disease resistance. Moreover, the genotyping information was employed to assess population genetics and structure measures. This work was framed in the TREASURE project (https://treasure.kis.si), a multidisciplinary European Union funded project pointing toward development of sustainable pork chains in several European local pig breeds.

## Material and methods

Blood samples were obtained from 47 individuals from each one of the 20 breeds included in the study: Black Slavonian and Turopolje (Croatia), Basque and Gascon (France), Schwäbisch-Hällisches Schwein (Germany), Apulo-Calabrese, Casertana, Cinta Senese, Mora Romagnola, Nero Siciliano and Sarda (Italy), Lithuanian indigenous wattle and Lithuanian White old type (Lithuania), Alentejana and Bísara (Portugal), Moravka and Swallow-Bellied Mangalitsa (Serbia), Krškopolje (Slovenia) and Iberian and Majorcan Black (Spain). Selection of individuals for sampling was performed avoiding highly related animals (no full- or half-sibs), balancing between sexes and prioritizing adult individuals or at least animals with adult morphology. Specialized professionals from each institution that provided animal material obtained all blood samples following standard routine monitoring procedures and guidelines, at farm or at slaughter. No animal experiment was performed within this research and blood samples were obtained as a general breeding procedure and only reused here.

The genomic DNA was extracted from leukocytes present in 8–15 mL of peripheral blood, collected in Vacutainer tubes containing 10% 0.5 M EDTA (ethylenediaminetetraacetic acid, disodium dihydrate salt) at pH 8.0. The extraction was performed using either a standardized phenol-chloroform, high-salt method or a commercial kit [4].Most interesting candidate genes for relevant productive traits in pigs were prioritized among those prone to be genotyped with TaqMan medium-throughput OpenArray Genotyping platform (Thermo Fisher Scientific). For the SNP selection, previous evidences of association with relevant traits (morphological, productive, reproductive, meat quality and disease resistance traits) were considered, prioritizing specially those which seemed to be potential or known causal mutations. A final SNP panel of 39 SNPs (Table 1) was selected and genotyped by the aforementioned OpenArray system. The genotyping was performed in a QuantStudio 12 K Flex Real-Time PCR System (Thermo Fisher Scientific) at the Centre for Research in Agricultural Genomics (CRAG, Barcelona, Spain). In this procedure, SNPs are typed using TaqMan genotyping chemistry supported on a metal-based array. DNA samples were loaded and amplified on arrays as recommended by the manufacturer. Endpoint detection of signal intensities of allele specific fluorescent dyes was conducted by the OpenArray NT Imager, and genotypes were called using the OpenArray SNP Genotyping analysis software. All SNPs were visually examined for any clustering issues.

**Table 1:** SNP selection and their association with quantitative traits.

Gene	SSC	Genome position	Polymorphism	Associated Traits	References
***ACACA***	12	38624687	c.5634T>C	Fatness, meat quality, FA profile	[14]
***ACSL4***	X	89763094	c.2645G>A	Meat quality, FA profile	[15]
***ADIPOQ***	13	124643017	c.1735G>A	Fatness	[16]
***AHR***	9	86550830	c.2149G>T	Litter size	[17]
***CAPNS1***	6	45514212	c.429A>C	Meat quality	[18]
***CAST***	2	103299934	g.103299934G>A	Meat quality	[19]
***CYB5A***	1	149737752	g.149737752G>T	Meat quality, boar taint	[20,21]
***CYP2E1***	14	141690107	g. 141690107C>T	Meat quality, boar taint	[22,23]
***ESR1***	1	14418777	c.669T > C	Litter size	[24]
***FASN***	12	926299	g.926299G>A	Fatness, meat quality, FA profile	[25]
***FTO***	6	31460242	g. 31460242A>T	Growth, fatness	[26]
***FUT1***	6	54079560	g.54079560T>C	Disease resistance	[27]
***GBP5***	4	127301202	g.127301202G>T	Disease resistance	[28–30]
***IGF2***	2	1483832	g. 1483832G>A	Growth, fatness	[31]
***KIT***	8	41488472	g. 41488472C>T	Coat color	[32–34]
***LEP***	18	20111759	g.20111759A>G	Growth, fatness	[35]
***LEPR***	6	146829589	c.1987C>T	Growth, fatness	[36,37]
***MC1R***	6	181461	g.181461G>A	Coat color	[38,39]
***MC1R***	6	181697	g.181697C>T	Coat color	[38,39]
***MC1R***	6	181818	g.181818G>A	Coat color	[38,39]
***MC1R***	6	181825	g.181825T>C	Coat color	[38,39]
***MC1R***	6	181905	g. 181905G>A	Coat color	[38,39]
***MC1R***	6	182126	g.182126CC>*	Coat color	[38,39]
***MC4R***	1	160773437	c.892G>A	Growth, fatness	[40]
***MSTN***	15	94629248	g.94629248C>T	Growth, fatness	[41]
***MTTP***	8	120821998	c.2573T>C	Meat quality, FA profile	[42]
***MUC4***	13	134226654	g. 134226654C>G	Disease resistance	[43]
***NR6A1***	1	265347265	c. 748T>C	Number of vertebrae	[44]
***PCK1***	17	57932233	c.2456A>C	Meat quality	[45]
***PHKG1***	3	16830320	g. 16830320C>A	Fatness, meat quality	[46]
***PPARD***	7	31281804	g.31281804G>A	Ear size	[47]
***PPARGC1A***	8	17867068	c.1288T>A	Meat quality	[18]
***PRKAG3***	15	120863537	g. 120863537G>A	meat quality	[48,49]
***PRKAG3***	15	120863533	g. 120863533G>A	meat quality	[48,49]
***RYR1***	6	47357966	c.1843C>T	performance, meat quality	[50,51]
***SCD***	14	111461751	g.111461751C>T	Meat quality, FA profile	[52]
***TAS2R39***	18	7068883	g.7068883T>G	Fatness	[53]
***TAS2R4***	UN	8135115	g. 8135115A>T	Fatness	[53]
***TYRP1***	1	209733431	g.209733431A>G	Coat color	[54]

Polymorphism locations are indicated according to Sus Scrofa genome annotation v11.1

Melanocortin receptor 1 (*MC1R*) haplotypes were constructed from the data on individual SNPs located at the following positions on SSC6 according to Ss11.1: g.181461 (G>A; p.A243T)– g.181697 (C>T; p.A164V)– g.181818 (G>A; p.D124N)– g.181825 g.181825 (T>C; p.L102P)– g.181905 (G>A; p.V95M)– g.182126 (CC>*), with the following definition of haplotypes: MC1R*1: GCGTG**; MC1R*2: GCGCA**; MC1R*3: GCATG**; MC1R*4: ATGTG**; MC1R*6: GCATGCC; MC1R*7: GCGTGCC

Genetic variability at the different loci in each population was measured with observed (H_O_) and expected (H_E_) heterozygosity and F_ST_ [5]. Population structure was evaluated using the F_IS_ statistic. D_S_ and F_ST_ distance measures were estimated according to Takezaki and Nei [6]. These estimates and allele frequencies were computed using the *hierfstat* library in R environment [7]. Neighbor-joining trees were constructed using D_S_ and F_ST_ distances with the *nj* function belonging to the *ape* library in R [8]. Bootstrap re-sampling (n = 1,000) was performed to test the robustness of the phylogenetic tree topologies.

The population structure was also analyzed by a Discriminant Analysis of Principal Component (DAPC) through *adegenet* package v.2.0.2 [9] in R environment [10]. The optimal number of clusters was identified through the Bayesian Information Criterion (BIC) and clusters were plotted in a scatterplot of the first and second linear discriminants of DAPC. Assignment of the individuals at clusters was plotted using the results coming from two different methods: α-score optimization and cross-validation. Both methods provide the optimal number of principal components (PC) to retain finding a trade-off between power of discrimination and overfitting. For the cross-validation, data were randomly divided in a training set (90%) and a validation set (10%) with 1000 replications. Members of each group were selected in order to ensure that each population in the original data set was represented in both training and validation sets. The optimal number of PCs to retain was based on the lowest root mean square error.

The most likely number of partitions in the dataset, irrespective of breed of origin, was also determined using the algorithm implemented in STRUCTURE [11]. We carried out 20 different runs from K = 1 to K = 25. All runs used a burn-in period of 50,000 iterations and a period of data collection of 200,000 iterations under an admixture model with allele frequencies correlated. The most likely number of clusters (K) was chosen following Evanno *et al*. [12] recommendations. The similarity of the outcomes of the 20 solutions was assessed using CLUMMP software [13]; the most frequent solution was considered to be the most probable.

## Results and discussion

The analyzed SNP panel includes polymorphisms related to coat color and morphological traits, growth, fatness and carcass traits, meat quality traits, reproduction and disease resistance.

TaqMan allelic discrimination qPCR assays have been shown to be accurate and reliable [55,56]. The initial validation of the assays showed the successful genotyping of all the tested mutations. Unique clusters indicative of different genotypes were formed based on the signal intensity ratio of the employed probes for 36 out of the 39 analyzed SNPs. The remaining three SNPs were monomorphic showing only one cluster. The success rate of each genotyped marker (call rate) was higher than 98%. Regarding the samples, those with genotyping call rates lower than 80% were discarded, resulting in a successful genotyping for 95% of the analyzed samples. The number of animals successfully genotyped in each breed and allele frequencies are shown in Tables 2–5. Genotyping data is included in S1 Table.

**Table 2:** Allele frequencies for seven haplotypes and four polymorphisms on candidate genes for coat colour and morphological traits, in 20 European local breeds.

Breed (n)	*MC1R*^†^						*KIT*		*TYRP1*		*NR6A1*		*PPARD*	
	1	2	3	4	6	7	C	T	A	G	T	C	G	A
Alentejana (47)	0.00	0.01	0.09	0.00	0.66	0.24	**1.00**	**0.00**	**1.00**	**0.00**	0.85	0.15	**1.00**	**0.00**
Apulo-Calabrese (44)	0.00	0.00	0.90	0.00	0.10	0.00	0.99	0.01	**1.00**	**0.00**	0.80	0.20	**1.00**	**0.00**
Basque (47)	0.00	0.01	0.98	0.00	0.01	0.00	0.88	0.12	**1.00**	**0.00**	**1.00**	**0.00**	**1.00**	**0.00**
Bísara (47)	0.00	0.00	0.86	0.01	0.13	0.00	**1.00**	**0.00**	**1.00**	**0.00**	**1.00**	**0.00**	0.83	0.17
Black Slavonian (30)	0.00	0.89	0.04	0.02	0.06	0.00	0.99	0.01	**1.00**	**0.00**	0.92	0.08	0.92	0.08
Casertana (46)	0.00	0.00	0.89	0.02	0.09	0.00	0.98	0.02	0.98	0.02	0.98	0.02	0.95	0.05
Cinta Senese (46)	0.00	0.00	0.78	0.06	0.16	0.00	0.02	0.98	**1.00**	**0.00**	**1.00**	**0.00**	**1.00**	**0.00**
Gascon (47)	0.00	0.00	0.98	0.00	0.02	0.00	**1.00**	**0.00**	0.99	0.01	**1.00**	**0.00**	**1.00**	**0.00**
Iberian (47)	0.00	0.00	0.08	0.00	0.66	0.26	**1.00**	**0.00**	**1.00**	**0.00**	0.88	0.13	**1.00**	**0.00**
Krškopolje (36)	0.00	0.00	0.82	0.02	0.16	0.00	0.98	0.02	0.95	0.05	**1.00**	**0.00**	0.87	0.13
Lithuanian indigenous wattle (47)	0.00	0.00	0.00	0.13	0.87	0.00	**1.00**	**0.00**	**1.00**	**0.00**	0.61	0.39	0.82	0.18
Lithuanian White Old Type(47)	**0.00**	**0.00**	**0.00**	**0.00**	**1.00**	**0.00**	0.97	0.03	**1.00**	**0.00**	0.99	0.01	0.99	0.01
Majorcan Black (47)	0.00	0.02	0.92	0.01	0.05	0.00	0.88	0.12	**1.00**	**0.00**	0.95	0.05	**1.00**	**0.00**
Mora Romagnola (43)	0.52	0.00	0.00	0.48	0.00	0.00	**1.00**	**0.00**	**1.00**	**0.00**	**1.00**	**0.00**	**1.00**	**0.00**
Moravka (47)	0.03	0.37	0.43	0.00	0.17	0.00	0.79	0.21	0.82	0.18	**1.00**	**0.00**	0.94	0.06
Nero Siciliano(38)	0.01	0.04	0.75	0.03	0.18	0.00	0.99	0.01	0.99	0.01	0.73	0.27	0.96	0.04
Sarda (46)	0.01	0.04	0.26	0.03	0.66	0.00	0.86	0.14	0.99	0.01	0.98	0.02	0.98	0.02
Schwäbisch-Hällisches Schwein (46)	0.00	0.16	0.84	0.00	0.00	0.00	0.83	0.17	**1.00**	**0.00**	**1.00**	**0.00**	0.96	0.04
Swallow-Bellied Mangalitsa (46)	**1.00**	**0.00**	**0.00**	**0.00**	**0.00**	**0.00**	0.21	0.79	**1.00**	**0.00**	0.98	0.02	0.92	0.08
Turopolje (45)	**0.00**	**0.00**	**0.00**	**0.00**	**1.00**	**0.00**	**1.00**	**0.00**	**1.00**	**0.00**	0.57	0.43	0.99	0.01

^**†**^MC1R haplotypes: MC1R*1: GCGTG**; MC1R*2: GCGCA**; MC1R*3: GCATG**; MC1R*4: ATGTG**; MC1R*6: GCATGCC; MC1R*7: GCGTGCC.; Fixed alleles in bold.

**Table 3:** Allele frequencies for nine polymorphisms on candidate genes for meat production and carcass traits, in 20 European local breeds.

Breed (n)	*RYR 1*		*IGF2*		*MC4R*		*LEPR*		*LEP*		*FTO*		*MSTN*		*TAS2R39*		*TAS2R4*	
	T	C	A	G	G	A	C	T	C	T	A	T	A	G	T	G	A	T
Alentejana (47)	**0.00**	**1.00**	**0.00**	**1.00**	0.98	0.02	0.02	0.98	0.16	0.84	0.32	0.68	0.80	0.20	0.01	0.99	**1.00**	**0.00**
Apulo-Calabrese (44)	0.02	0.98	0.85	0.15	0.57	0.43	0.71	0.29	0.66	0.34	0.12	0.88	0.20	0.80	0.04	0.96	**1.00**	**0.00**
Basque (47)	**0.00**	**1.00**	**0.00**	**1.00**	**1.00**	**0.00**	**1.00**	**0.00**	0.27	0.73	0.84	0.16	0.39	0.61	0.00	1.00	**1.00**	**0.00**
Bísara (47)	0.05	0.95	0.01	0.99	0.94	0.06	0.74	0.26	0.94	0.06	0.50	0.50	0.81	0.19	0.13	0.87	**1.00**	**0.00**
Black Slavonian (30)	**0.00**	**1.00**	0.08	0.92	0.94	0.06	0.62	0.38	0.62	0.38	0.21	0.79	0.11	0.89	0.21	0.79	**1.00**	**0.00**
Casertana (46)	0.09	0.91	0.03	0.97	0.81	0.19	0.89	0.11	0.66	0.34	0.51	0.49	0.86	0.14	0.01	0.99	**1.00**	**0.00**
Cinta Senese (46)	0.01	0.99	0.12	0.88	0.29	0.71	0.87	0.13	0.47	0.53	0.33	0.67	0.66	0.34	0.27	0.73	**1.00**	**0.00**
Gascon (47)	0.03	0.97	0.00	1.00	0.33	0.67	0.88	0.13	0.48	0.52	0.50	0.50	0.92	0.08	**0.00**	**1.00**	**1.00**	**0.00**
Iberian (47)	**0.00**	**1.00**	0.06	0.94	0.95	0.05	**0.00**	**1.00**	0.26	0.74	0.25	0.75	0.95	0.05	0.06	0.94	**1.00**	**0.00**
Krškopolje (36)	0.21	0.79	0.39	0.61	0.34	0.66	0.60	0.40	0.77	0.23	0.34	0.66	0.57	0.43	0.23	0.77	**1.00**	**0.00**
Lithuanian indigenous wattle (47)	0.09	0.91	0.10	0.90	0.38	0.63	0.41	0.59	0.85	0.15	0.04	0.96	0.34	0.66	0.01	0.99	**1.00**	**0.00**
Lithuanian White Old Type (47)	**0.00**	**1.00**	0.28	0.72	0.46	0.54	0.82	0.18	**1.00**	**0.00**	0.26	0.74	0.75	0.25	0.09	0.91	**1.00**	**0.00**
Majorcan Black (47)	**0.00**	**1.00**	0.19	0.81	0.94	0.06	0.34	0.66	0.17	0.83	0.31	0.69	0.75	0.25	0.42	0.58	**1.00**	**0.00**
Mora Romagnola (43)	**0.00**	**1.00**	0.10	0.90	0.82	0.18	**1.00**	**0.00**	0.26	0.74	0.82	0.18	0.99	0.01	0.01	0.99	**1.00**	**0.00**
Moravka (47)	0.02	0.98	0.26	0.74	0.54	0.46	0.58	0.42	0.63	0.37	0.35	0.65	0.39	0.61	0.02	0.98	**1.00**	**0.00**
Nero Siciliano (38)	0.01	0.99	0.20	0.80	0.63	0.17	0.89	0.11	0.55	0.45	0.39	0.61	0.49	0.51	0.18	0.82	**1.00**	**0.00**
Sarda (46)	0.05	0.95	0.27	0.73	0.63	0.37	0.36	0.64	0.47	0.53	0.38	0.62	0.79	0.21	0.29	0.71	**1.00**	**0.00**
Schwäbisch-Hällisches Schwein (46)	**0.00**	**1.00**	0.50	0.50	0.70	0.30	0.95	0.05	0.64	0.36	0.75	0.25	0.43	0.57	0.09	0.91	**1.00**	**0.00**
Swallow-Bellied Mangalitsa (46)	**0.00**	**1.00**	**0.00**	**1.00**	0.93	0.07	0.76	0.24	0.02	0.98	0.37	0.63	0.49	0.51	0.17	0.83	**1.00**	**0.00**
Turopolje (45)	**0.00**	**1.00**	**0.00**	**1.00**	0.87	0.13	**1.00**	**0.00**	0.35	0.65	0.95	0.05	0.72	0.28	**0.00**	**1.00**	**1.00**	**0.00**

Fixed alleles in bold

**Table 4:** Allele frequencies for 15 polymorphisms on candidate genes for meat quality, in 20 European local breeds.

Breed (n)	*SCD*		*ACACA*		*ACSL4*		*FASN*		*MTTP*		*CYP2E1*		*CYB5A*		*CAST*		*PPARGC1A*		*CAPNS1*		*ADIPOQ*		*PRKAG3 I199V*		*PRKAG3 R200Q*		*PHKG1*		*PCK1*	
	T	C	C	T	G	A	G	A	C	T	C	T	G	T	G	A	T	A	A	C	G	A	G	A	G	A	C	A	A	C
Alentejana (47)	**1.00**	**0.00**	0.44	0.56	0.46	0.54	0.17	0.83	0.54	0.46	0.98	0.02	0.50	0.50	0.20	0.80	0.03	0.97	0.45	0.55	0.98	0.02	0.30	0.70	**1.00**	**0.00**	**1.00**	**0.00**	0.97	0.03
Apulo-Calabrese (44)	0.74	0.26	0.63	0.37	0.71	0.29	0.53	0.47	0.47	0.53	0.83	0.17	0.99	0.01	0.68	0.32	0.63	0.37	0.19	0.81	**1.00**	**0.00**	0.50	0.50	**1.00**	**0.00**	0.50	0.50	0.67	0.33
Basque (47)	0.97	0.03	0.81	0.19	0.99	0.01	0.34	0.66	0.07	0.93	**1.00**	**0.00**	0.91	0.09	0.35	0.65	0.68	0.32	0.13	0.88	0.98	0.02	0.71	0.29	**1.00**	**0.00**	**1.00**	**0.00**	0.50	0.50
Bísara (47)	0.65	0.35	0.64	0.36	0.86	0.14	0.46	0.54	0.15	0.85	0.56	0.44	0.73	0.27	0.67	0.33	0.40	0.60	0.52	0.48	0.90	0.10	0.41	0.59	**1.00**	**0.00**	0.94	0.06	0.52	0.48
Black Slavonian (30)	0.50	0.50	0.71	0.29	0.11	0.89	0.52	0.48	0.44	0.56	0.87	0.13	0.94	0.06	0.56	0.44	0.63	0.37	0.42	0.58	0.58	0.42	0.73	0.27	**1.00**	**0.00**	**1.00**	**0.00**	0.52	0.48
Casertana (46)	0.73	0.27	0.42	0.58	0.91	0.09	0.58	0.42	0.44	0.56	0.82	0.18	0.98	0.02	0.68	0.32	0.48	0.52	0.71	0.29	**1.00**	**0.00**	0.14	0.86	**1.00**	**0.00**	0.97	0.03	0.33	0.67
Cinta Senese (46)	0.96	0.04	0.83	0.17	0.36	0.64	0.59	0.41	0.91	0.09	0.54	0.46	0.69	0.31	0.32	0.68	0.97	0.03	0.18	0.82	**1.00**	**0.00**	0.66	0.34	**1.00**	**0.00**	0.96	0.04	0.54	0.46
Gascon (47)	0.91	0.09	0.39	0.61	0.97	0.03	0.31	0.69	0.44	0.56	0.21	0.79	0.95	0.05	0.43	0.57	0.56	0.44	0.18	0.82	**1.00**	**0.00**	0.69	0.31	**1.00**	**0.00**	**1.00**	**0.00**	0.15	0.85
Iberian (47)	0.98	0.02	0.48	0.52	0.65	0.35	0.43	0.58	0.77	0.23	0.95	0.05	0.51	0.49	0.14	0.86	0.06	0.94	0.61	0.39	0.96	0.04	0.36	0.64	**1.00**	**0.00**	0.95	0.05	0.96	0.04
Krškopolje (36)	0.85	0.15	0.60	0.40	0.73	0.27	0.68	0.32	0.37	0.63	0.45	0.55	0.98	0.02	0.91	0.09	0.37	0.63	0.19	0.81	0.79	0.21	0.78	0.22	**1.00**	**0.00**	0.58	0.42	0.34	0.66
Lithuanian indigenous wattle (47)	0.64	0.36	0.97	0.03	0.85	0.15	0.52	0.48	0.41	0.59	0.84	0.16	0.81	0.19	0.45	0.55	0.57	0.43	0.73	0.27	0.98	0.02	0.50	0.50	**1.00**	**0.00**	0.44	0.56	0.16	0.84
Lithuanian White Old Type (47)	0.74	0.26	0.79	0.21	0.15	0.85	0.50	0.50	0.33	0.67	0.70	0.30	0.83	0.17	0.74	0.26	0.14	0.86	0.36	0.64	0.85	0.15	0.93	0.07	**1.00**	**0.00**	0.95	0.05	0.57	0.43
Majorcan Black (47)	0.95	0.05	0.47	0.53	0.60	0.40	0.35	0.65	0.60	0.40	0.90	0.10	0.82	0.18	0.80	0.20	0.27	0.73	0.60	0.40	0.99	0.01	0.56	0.44	**1.00**	**0.00**	**1.00**	**0.00**	0.91	0.09
Mora Romagnola (43)	0.17	0.83	0.33	0.67	0.93	0.07	0.55	0.45	0.52	0.48	0.53	0.47	0.94	0.06	0.19	0.81	0.84	0.16	0.88	0.12	0.89	0.11	0.89	0.11	**1.00**	**0.00**	0.98	0.02	0.89	0.11
Moravka (47)	0.85	0.15	0.55	0.45	0.54	0.46	0.42	0.58	0.45	0.55	0.36	0.64	0.95	0.05	0.71	0.29	0.46	0.54	0.44	0.56	0.88	0.12	0.53	0.47	**1.00**	**0.00**	0.68	0.32	0.78	0.22
Nero Siciliano (38)	0.94	0.06	0.57	0.43	0.68	0.32	0.36	0.64	0.46	0.54	0.54	0.46	0.91	0.09	0.50	0.50	0.61	0.39	0.50	0.50	0.94	0.06	0.88	0.12	**1.00**	**0.00**	0.98	0.02	0.54	0.46
Sarda (46)	0.94	0.06	0.65	0.35	0.65	0.35	0.58	0.42	0.37	0.63	0.65	0.35	0.83	0.17	0.69	0.31	0.64	0.36	0.47	0.53	0.92	0.08	0.61	0.39	**1.00**	**0.00**	0.58	0.42	0.55	0.45
Schwäbisch-Hällisches Schwein (46)	0.89	0.11	0.47	0.53	0.89	0.11	0.28	0.72	0.13	0.87	0.80	0.20	**1.00**	**0.00**	0.79	0.21	0.47	0.53	0.26	0.74	0.92	0.08	0.60	0.40	**1.00**	**0.00**	**1.00**	**0.00**	0.31	0.69
Swallow-Bellied Mangalitsa (46)	0.97	0.03	0.77	0.23	0.64	0.36	0.57	0.43	0.19	0.81	0.90	0.10	0.99	0.01	0.88	0.12	0.56	0.44	0.78	0.22	**1.00**	**0.00**	0.15	0.85	**1.00**	**0.00**	**1.00**	**0.00**	**1.00**	**0.00**
Turopolje (45)	**1.00**	**0.00**	0.29	0.71	0.56	0.44	0.51	0.49	0.37	0.63	0.36	0.64	**1.00**	**0.00**	0.78	0.22	0.03	0.97	**0.00**	**1.00**	**1.00**	**0.00**	0.19	0.81	**1.00**	**0.00**	**1.00**	**0.00**	0.09	0.91

Fixed alleles in bold

**Table 5:** Allele frequencies for five polymorphisms on candidate genes for disease resistance and prolificacy in 20 European local breeds.

Breed (n)	*GBP5*		*MUC4*		*FUT1*		*ESR1*		*AHR*	
	G	T	C	G	A	G	G	A	G	T
Alentejana (47)	0.98	0.02	**1.00**	**0.00**	0.07	0.93	**1.00**	**0.00**	0.05	0.95
Apulo-Calabrese (44)	0.88	0.12	0.87	0.13	0.04	0.96	**1.00**	**0.00**	0.55	0.45
Basque (47)	**1.00**	**0.00**	0.91	0.09	0.04	0.96	**1.00**	**0.00**	0.32	0.68
Bísara (47)	0.78	0.22	0.60	0.40	0.47	0.53	**1.00**	**0.00**	0.26	0.74
Black Slavonian (30)	0.88	0.12	0.83	0.17	0.43	0.57	**1.00**	**0.00**	0.52	0.48
Casertana (46)	0.88	0.12	0.81	0.19	0.51	0.49	**1.00**	**0.00**	0.64	0.36
Cinta Senese (46)	0.49	0.51	0.94	0.06	0.12	0.88	**1.00**	**0.00**	0.49	0.51
Gascon (47)	0.82	0.18	0.82	0.18	0.48	0.52	**1.00**	**0.00**	0.27	0.73
Iberian (47)	0.92	0.08	0.95	0.05	0.06	0.94	**1.00**	**0.00**	0.07	0.93
Krškopolje (36)	0.95	0.05	0.84	0.16	0.71	0.29	**1.00**	**0.00**	0.44	0.56
Lithuanian indigenous wattle (47)	0.43	0.57	0.47	0.53	0.25	0.75	**1.00**	**0.00**	0.50	0.50
Lithuanian White Old type (47)	0.87	0.13	0.59	0.41	0.09	0.91	**1.00**	**0.00**	0.80	0.20
Majorcan Black (47)	0.91	0.09	0.88	0.12	0.25	0.75	**1.00**	**0.00**	0.11	0.89
Mora Romagnola (43)	0.58	0.42	**1.00**	**0.00**	0.09	0.91	**1.00**	**0.00**	0.13	0.88
Moravka (47)	0.93	0.07	0.89	0.11	0.09	0.91	**1.00**	**0.00**	0.42	0.58
Nero Siciliano (38)	0.84	0.16	0.89	0.11	0.12	0.88	**1.00**	**0.00**	0.24	0.76
Sarda (46)	0.73	0.27	0.77	0.23	0.18	0.82	**1.00**	**0.00**	0.48	0.52
Schwäbisch-Hällisches Schwein (46)	0.96	0.04	0.76	0.24	0.41	0.59	**1.00**	**0.00**	0.50	0.50
Swallow-Bellied Mangalitsa (46)	0.77	0.23	0.79	0.21	0.21	0.79	**1.00**	**0.00**	0.20	0.80
Turopolje (45)	0.93	0.07	0.68	0.32	0.74	0.26	**1.00**	**0.00**	0.48	0.52

Fixed alleles in bold

### Markers related to morphological and coat color traits

**Fig 1:**
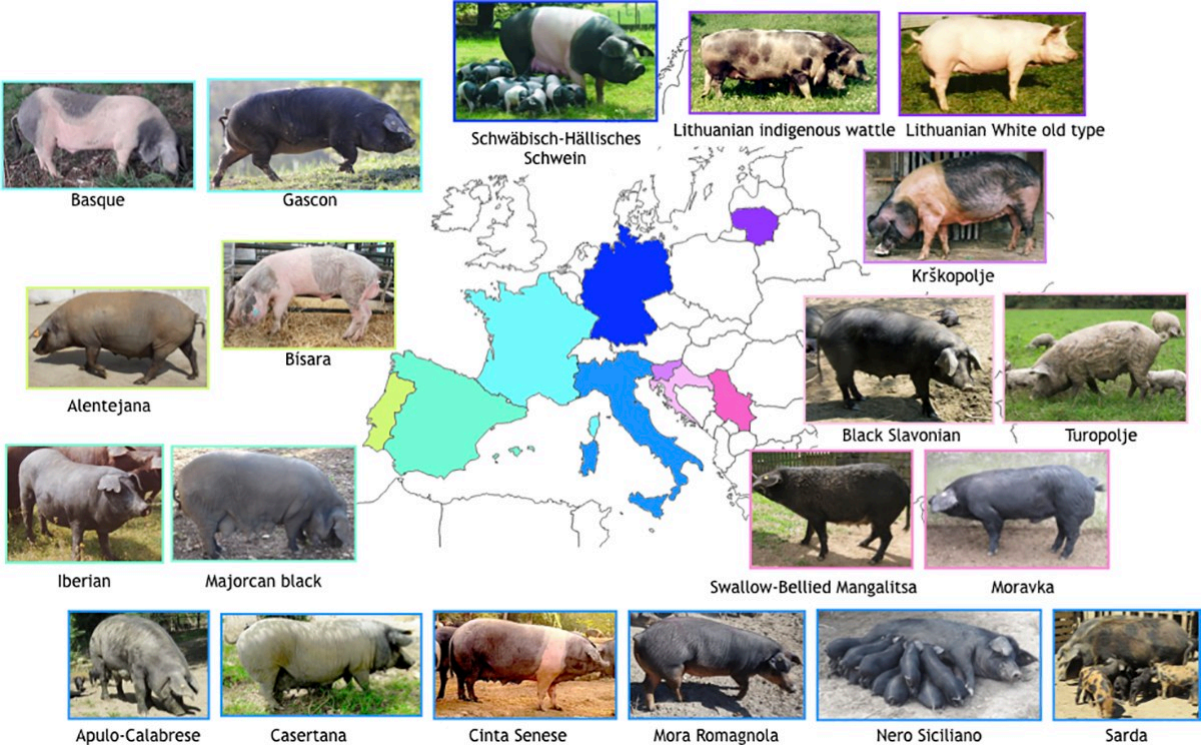
Phenotype and geographical origin of the 20 analysed pig breeds: Black Slavonian and Turopolje (Croatia), Basque and Gascon (France), Schwäbisch-Hällisches Schwein (Germany), Apulo-Calabrese, Casertana, Cinta Senese, Mora Romagnola, Nero Siciliano and Sarda (Italy), Lithuanian indigenous wattle and Lithuanian White old type (Lithuania), Alentejana and Bísara (Portugal), Moravka and Swallow-Bellied Mangalitsa (Serbia), Krškopolje (Slovenia) and Iberian and Majorcan Black (Spain).

A picture showing the characteristic phenotype and country of origin for each breed is shown in Fig 1. In general, low segregation was observed for markers in genes involved in coat color and morphology traits (Table 2), as it could be expected as these are the main selection criteria employed by breeders over the years, even in local breeds. In pigs, a large variety of coat colors and patterns, characteristics of different breeds and populations, have been produced following domestication and selection [57–59]. Among the several loci involved in pigmentation, *MC1R* and v-kit Hardy-Zuckerman 4 feline sarcoma viral oncogene homolog gene (*KIT*) genes are supposed to play major roles in determining coat color variation in Mediterranean pig breeds or populations [60].

*MC1R* gene codes for a G protein-coupled receptor that is primarily expressed in melanocytes and plays a key role in melanogenesis. It is involved in regulation of eumelanin (black/brown) and phaeomelanin (yellow/red) synthesis in the melanocyte and is encoded by the Extension (*E*) coat color locus. Several SNPs were genotyped for *MC1R* locus, which were used for the construction of haplotypes [38,59]. Five main haplotypes are usually considered. The wild allele (MC1R*1, *E*^*+*^), which in many species involves synthesis of both melanin types and is present in wild boars, was present in Mora Romagnola (0.52) and fixed in Swallow-Bellied Mangalitsa. The fixation of MC1R*1 in Swallow-Bellied Mangalitsa breed has already been reported [59]. Moreover, this breed was proposed as the only domestic breed carrying this allele among a wide panel of European and Chinese commercial and local breeds [59]. Nevertheless, we also detect a very small introgression of MC1R*1 in Moravka (≤0.03), Nero Siciliano and Sarda (≤0.01). The MC1R*2 allele (E^D1^), characterized by a p.L102P aminoacid change is associated with dominant black coat. This allele has Asian origin and has been previously detected in Chinese and Large Black breeds. According to our results MC1R*2 is observed at high frequency in Black Slavonian (0.89) and with moderate presence in Moravka and Schwäbisch-Hällisches. The Black Slavonian pig comes from eastern Croatia and was created at the end of the 19th century by the crossbreeding of Swallow-Bellied Mangalitsa, Berkshire, Large Black and Poland China breeds [61,62], which explains the origin of the Asian *MC1R* allele. Interestingly, MC1R*1 allele probably provided by Swallow-Bellied Mangalitsa has not persisted, possibly due to selection of the black coat color. Since the Black Slavonian pig is the only Croatian breed with MC1R*2 genotype, the genotyping of this locus has been already proposed to determine the purity of the Black Slavonian pigs [62], as uncontrolled crossing with modern pig breeds is usual. According to our results, some introgression of alleles 3, 4 and 6 is observed in Black Slavonian, which may indicate contamination with commercial breeds such as Duroc, Pietrain or Large White.

Haplotypes MC1R*3 (*E*^*D2*^) and MC1R*6 (*E*^*P*^), are widely observed in European breeds. Allele MC1R*3 is associated with a p.D124N substitution involving black coat color. Allele MC1R*6 causes black spotting on a red or white background and possesses, in addition to the p.D124N substitution for dominant black coloring, a two base pair insertion at codon 22. These two alleles are predominant in most of the analyzed breeds, which is in agreement with the dark coat color of most of them. MC1R*3 mutation is the most frequent in Mediterranean and Pyrenean pig breeds whereas this variant is practically absent in Iberian Peninsula breeds. MC1R*6 allele showed the highest frequency in Lithuanian indigenous wattle and Turopolje breeds in agreement with its characteristic patched phenotype. This allele was also fixed in Lithuanian White Old Type, in which the white coat color must be consequence of variation in the *KIT* locus.

At last, allele MC1R*4 (e), associated with the recessive red coat color and characteristic of the Duroc breed was present in Mora Romagnola and detected at very low frequency in several other breeds. This allele was previously indicated to be fixed in the Mora Romagnola breed [63], which shows a solid phaeomelanic coat color. However, according to our results allele MC1R*4 is not fixed in this Italian breed but sharing distribution with MC1R*1 allele (allele frequencies 0.48 and 0.52, respectively). The small introgression of MC1R*4 allele in other breeds may be caused by crossbreeding with Duroc, which is a usual strategy in various breeds to improve growth rate and feed efficiency. For example, the use of Italian Duroc has been proposed to improve the farming performance of Cinta Senese without reducing the fresh meat quality [64] and crossbreeding with Duroc has also been evaluated in Lithuanian indigenous wattle showing increased performance [65]. Although crossbreeding with Duroc is the main production system of Iberian pigs, no introgression is observed in the pure Iberian genetic pool.

We previously detected a new allele, MC1R*7 [66], which was only present in the closely related Iberian and Alentejana breeds. This allele was characterized by the combination of the CC insertion without the p.D124N substitution. To the best of our knowledge, this combination has only been detected in a European pig with unknown phenotype [67]and in Iberian pigs with red coat color [66].

*KIT* gene plays key roles in melanogenesis, erythropoiesis, spermatogenesis and T-cell differentiation. Jointly with *MC1R*, they are the major determinants of the across-breed differences in coat color patterns in different mammals. Mutations in the porcine *KIT* gene (dominant white locus) have been shown to affect coat color and color distribution [66]. A large number of alleles have been reported in pigs [33,34] but their differentiation is complex as some are associated to copy number variation (CNV) of the gene, with a duplication or a triplication or even a higher number of copies of a region including the whole coding sequence, besides a splicing mutation, a 4bp deletion and other mutations, and their multiple combinations. Thus, full characterization of *KIT* alleles was beyond the scope of this work. Nevertheless, the *KIT* g. 43597545C>T (g.41488472 in Sscrofa11.1) SNP was included in the panel, since it had been previously associated to the belted phenotype [68]. This SNP was proposed as suitable marker for differentiation of Cinta Senese pigs from other non-belted breeds and in agreement our results show a very high frequency of allele T in Cinta Senese (0.98), while most other breeds are fixed or almost fixed for the C allele. These results are similar to those reported by Ogorevc *et al*. (2017) [69] in eleven pig breeds, some of them coinciding with the ones analyzed here. Although other breeds with variable belted patterns such as Krškopolje and Schwäbisch-Hällisches Schwein are represented in the current study, our results show low frequency of the T allele, 0.02 and 0.17, respectively. These results agree with those observed by Ogorevc *et al*. [69] suggesting that unlike Cinta Senese, these SNP cannot be used to differentiate Krškopolje and Schwäbisch-Hällisches Schwein from other non-belted breeds. However, the present work evidences that Swallow-Bellied Mangalitsa breed also shows high frequency (0.79) of allele T, suggesting an introgression with Hampshire alleles due to crossbreeding.

Besides the main roles of *MC1R* and *KIT* genes on coat color, other genes have been associated to specific coat patterns. For example, several polymorphisms in the porcine Tyrosinase-related protein 1 (*TYRP1*) gene have been recently associated with the blond coat color in Liangshan pigs [54]. One of the described SNPs, TYRP1 g.209733431A>G, was included in our panel. Although individuals with blond coat color have been described for some of the studied breeds like Swallow-Bellied Mangalitsa [70], only Krškopolje (0.05) and Moravka (0.18) breeds showed some introgression of the blond allele (G) of *TYRP1* gene even if the blond phenotype was not present in these breeds. These two breeds have known introgression of different commercial breeds. In the case of Krškopolje, German Landrace, Pietrain and Duroc breeds were commonly used as terminal sires for many years. Regarding Moravka, the creation of this breed is known to be the result of unsystematic crossing of the ancient and extinct local breed, Šumadinka, with Berkshire [71], and also crossing with Yorkshire could have taken place at the beginning of the 20^th^ century. These influences could explain the presence of unexpected alleles.

The orphan nuclear receptor, germ cell nuclear factor (*NR6A1*) gene has been shown to affect vertebrae number in the pig. While wild boars show a fixed vertebrae number (19), most domesticated pig breeds show a vertebrae number varying between 21 and 23 [72]. Previous reports indicate that the c. 748T allele, associated with higher number of vertebrae, appeared to be fixed in most studied breeds whereas in wild boars the c. 748C allele is fixed [73]. In our results, NR6A1 T allele was predominant in agreement with previous works. Lithuanian indigenous wattle and Turopolje showed the highest introgression of the C allele (0.39 and 0.43, respectively), associated with a shorter carcass and this is in agreement with data obtained from carcass measurements within TREASURE project (personal communication; results not yet published). The highest frequency of the C allele in Turopolje breed, one of the oldest pig breeds in Europe created for production in local forests, could probably be related to a long history of contacts with wild boars, as well as, the absence of any major selection or crossbreeding program [74]. Also Alentejana, Apulo-Calabrese, Majorcan Black, Black Slavonian, Iberian and Nero Siciliano showed small introgression of the wild allele which could be originated by uncontrolled mating with wild boars (frequencies ranging from 0.05 to 0.27) as these breeds are reared mainly in free range systems. Vertebrae number is an important trait in pig production as it influences the size of important meat cuts such as the loin and also has an overall effect on carcass conformation. Moreover, segregation of markers leading to changes in carcass conformation results in increased carcass heterogeneity, thus selection of the favorable T allele would be highly advisable, especially in Lithuanian indigenous wattle, Turopolje and Nero Siciliano breeds.

A missense mutation (p.G32E) of Chinese origin in the gene encoding the peroxisome proliferator-activated receptor delta (*PPARD*) gene was shown to be a Quantitative Trait Nucleotide (QTN) affecting the ear size in pigs [47]. The *PPARD* gene has roles in skin and cartilage development besides affecting fat metabolism. The Chinese allele A, which increases ear size, is supposed to be absent in European breeds, but it has been detected in Large Black [47]. According to our results, the Chinese allele is in general scarce but present at low or moderate frequencies in several breeds including Bísara (0.17), Black Slavonian (0.08), Krškopolje (0.13), Lithuanian indigenous wattle (0.18) and Swallow-Bellied Mangalitsa (0.08). A small introgression of Large Black in local Lithuanian pigs was made before and after World War I [75], therefore ear shape and size typical for Large Black could be found among Lithuanian indigenous wattle pigs. Also, the introgression of Large Black in Black Slavonian breed is already known, as previously discussed, and could explain the presence of allele A in this breed. The presence of the Chinese allele in Bísara is also compatible with the likely crossbreeding between this and Chinese or English breeds [76].

### Markers related to meat production and carcass traits

Nine polymorphisms related to meat production and carcass traits were studied (Table 3).

Porcine stress syndrome (PSS) or Malignant hyperthermia is a condition that develops in homozygous animals carrying a single coding nucleotide substitution in the *RYR1* gene(c.1843C>T; p.R615C), upon exposure to halogenated anaesthetics [50]. The *RYR1* gene codes for the skeletal ryanodine receptor, a protein structurally involved in calcium channels controlling movements of Ca^2+^ from the sarcoplasmic reticulum to the cytosol. PSS is of concern to the worldwide swine industry from both welfare and an economic perspective as it results in losses during transport and very fast post-mortem pH decline causing denaturation of muscle proteins, water loss and formation of pale, soft, exudative meats (PSE) with reduced meat quality and commercial value [50]. Among local breeds involved in the present study, as in most commercial breeds [50], the c.1843T mutant allele is scarce since many initiatives have been carried out to eliminate this allele. However, a relevant frequency was found (0.21) in Krškopolje breed (Table 3). High incidence of this mutation in Krškopolje breed could have likely arisen due to crossing with German Landrace in times when the breed was cast out, as it was for many years the most used terminal line in the breed’s recent history and it has a quite important incidence of this mutation. Although the presence of this allele in the population is known for some time, no efforts have been made by the breeders to eliminate it [50,77].

The insulin-like growth factor 2 (*IGF2*) intron 3 g.3072G>A (g.1483832G>A in Sscrofa11.1) substitution has been identified as a QTN for a paternally-expressed QTL affecting muscle growth, fat deposition, and heart size [31]. Pigs carrying the paternal A allele have higher lean growth and lower backfat thickness than those carrying the G allele [78,79]. The mutant A allele is common in breeds subjected to strong selection for lean meat content and would be of Asian origin [80], being predominant in commercial breeds such as Landrace or Large-White that had been historically introgressed with Asian breeds. This A allele is in general scarce in the breeds analyzed in the present work in agreement with the lack of selection for lean growth. However, it is present at considerable frequencies in a few breeds, such as Apulo-Calabrese who showed the highest frequency of the mutant allele (0.85), followed by Schwäbisch-Hällisches Schwein (0.50) and Krškopolje (0.39). In a pilot study of TREASURE project using modelling with InraPorc^®^software [81], Apulo-Calabrese and Krškopolje (Schwäbisch-Hällisches Schwein was not included in the study) showed the highest protein deposition, which could be related to the high frequencies of the mutant allele observed in these populations. Overall, this incidence can be explained by introgression of alleles from commercial breeds.

Several genes encoding proteins with key roles in energy homeostasis were included in the SNP panel. These functionally related genes are involved in the melanocortin pathway that regulates feed intake and energy expenditure at hypothalamic level. The leptin (LEP) is the signaling molecule, produced in adipocytes, which informs the central nervous system about the increase in energy stores, unchaining an anorexigenic signal through its hypothalamic receptor (LEPR). The melanocortin receptor 4 (MC4R) is a downstream molecule involved in transmitting the LEP signal. The fat mass and obesity-associated protein (*FTO*) gene is also most likely involved in the regulation of energy balance and feed intake [82]. Thus, all these genes are candidates for a role in the variability of feed intake regulation, growth and fattening [37,83]. The SNPs selected for these genes have been proposed as useful markers for future breeding purposes, with different levels of evidence for causality. For example, the *LEPR* gene mutation has been related to several productive, fatness and meat quality traits in different genetic backgrounds [36,84–86] and it has been also associated with the hypothalamic expression of LEPR and downstream molecules [37], suggesting functionality of this particular variant. LEPR.c.1987T allele is systematically associated with higher fatness and feed intake and is fixed in the obese Iberian pig breed. Our results match this finding and moreover show that allele T is almost fixed in Alentejana (0.98), a very close breed with the same origin in the Iberian Peninsula. Besides these two close breeds, allele T is in general scarce in most of the remaining breeds except in Majorcan Black and Sarda, which show intermediate frequencies, opening selection possibilities to increase meat quality, because of its interesting association with intramuscular fat and fatty acid profile. However, effects on intake, growth and fatness should be evaluated and considered in each particular breed. It is interesting to highlight that the *LEPR* favorable T allele is completely absent in Basque, Mora Romagnola and Turopolje breeds; and at very low frequency in many other breeds, also characterized by a fat phenotype. Nevertheless, this results agrees with the polygenic nature of fatness and meat quality traits. Regarding the *MC4R* gene, different SNPs have been previously evaluated. Among them, the c.892G>A (p.D298N) is usually considered the most relevant one, being associated with variation in growth and fatness traits in most breeds and crosses, but with some discrepancies among studies [40,87,88]. In general, allele A is considered to be associated with a high feed intake and high lipid deposition, although this allele was absent (Basque) or at very low frequencies in many of our breeds (Alentejana, Bísara, Black Slavonian, Iberian, Majorcan Black and Swallow-Bellied Mangalitsa). In our study, c.892G>A SNP showed intermediate frequencies (0.30 to 0.70) in Apulo-Calabrese, Cinta Senese, Gascon, Krškopolje, both Lithuanian breeds, Moravka and Sarda, being susceptible for association studies with growth and fatness. The SNPs located in the *LEP* and *FTO* genes [35,89] are segregating at intermediate frequencies in most analyzed populations. These SNPs are most probably markers in linkage disequilibrium with the causal mutation, since their effect on production traits have not been established for all the studied breeds and thus the future validation of their effects is essential.

Myostatin (MSTN) is a member of transforming growth factor-β (TGF-β) superfamily. It is a negative regulator for both embryonic development and adult muscle homeostasis. Previous works have detected and analyzed three SNPs (g.435G>A, g.447A>G and g.879T>A), located in the *MSTN* promoter region [90], concluding a relationship between these markers and muscle development. These mutations have been proposed as causal because of their location coincident with transcription factor binding sites, and their effect on mRNA expression [90]. We selected for genotyping the g.435G>A polymorphism (g.94629248C>T in Sscrofa11.1), which co-segregates with g.447A>G in several breeds [90–92]. According to previous works, the g.435A and g.447G alleles have positive effects on total muscle production and a negative effect on fat deposition. With a few exceptions (Gascon, Iberian and Mora Romagnola, which show a very high frequency of g.435A allele responsible for muscle growth increase), intermediate frequencies were observed in most of our breeds, thus allowing further association studies to be conducted in order to support its association as in most of them the minor allele frequency was between 0.11 and 0.49.

Taste 2 Receptors (*TAS2R*) gene family encodes for receptors involved in taste perception and bitter sensing. Among them, *TAS2R39* and *TAS2R4* genes have been recently reported to be related to backfat thickness, probably as a consequence of different feed intake, due to different feed taste perception [53]. For *TAS2R39* gene, the missense mutation, p.N71T, was included in our panel. Allele G was predominant or fixed in most breeds although some others showed moderate to intermediate frequency of T allele (0.21 to 0.29), such as Majorcan Black, Cinta Senese, Black Slavonian, Krškopolje or Sarda, in which the SNP may be used in association studies. Such studies could deepen in the effects and causality of the marker and the potential mechanism for affecting fat deposition (voluntary feed intake). Finally, the marker in *TAS2R4* gene showed the A allele fixed in all breeds.

### Markers related to meat and fat quality

The fatty acid profile is one of the main determinants of animal product quality and is one of the distinguishing characteristics of high-valued Iberian meat products. Fatty acid profile has profound effects on the nutritional, sensorial and technological properties of meat [93]. Different candidate genes, mainly involved in lipogenesis, have been proposed to explain the variability of fatty acid content in animal tissues (Table 4). In most of the cases, the detected SNPs are probably neutral markers in linkage disequilibrium with the causal mutation, as it would be the case for fatty acid synthase (*FASN*), acetyl CoA carboxilase alpha (*ACACA*) and microsomal triglyceride transfer protein (*MTTP*) genes, included in our panel. The *ACACA* and *FASN* genes are involved in *de novo* lipogenesis, and mutations in these genes have been associated to MUFA and SFA content [25]. The missense c.2573T>C mutation in the porcine *MTTP* gene is highly associated with the fatty acid composition in the pig [42], influencing the percentages of oleic, palmitic and linoleic acids. The C allele (coding for Leucine) is also associated with an increased MTTP lipid transfer activity. For these mutations a high level of segregation is observed in most of the breeds. Association studies should be performed in order to validate the mutational effects on the different genetic backgrounds and evaluate their usefulness in breeding programs targeting the improvement of meat quality.

Mutations in the stearoyl CoA desaturase (*SCD*) and long-chain acyl-CoA synthetase (*ACSL4*) genes may be considered causal mutations of effects on meat fatty acid profile, specifically regarding MUFA content. The SCD is a very relevant enzyme because it catalyzes the desaturation of palmitic and stearic to palmitoleic and oleic acids. For this gene a mutation g.2228T>C (g.111461751C>T in Sscrofa11.1), positioned in the core sequence of several putative transcription factor binding sites, has been repeatedly associated to MUFA content in different genetic backgrounds [52,94]. There are several plausible mechanisms by which allele T enhances 18∶1/18∶0 ratio and, consequently, the proportion of monounsaturated to saturated fatty acids, increasing meat quality. ACSL4 protein catalyzes the formation of long-chain acyl-CoA from fatty acid, ATP and CoA, playing an important role in both lipid biosynthesis and fatty acid turnover. The mutation c.2645G>A SNP located in the 3' untranslated region has been associated with the percentages of oleic and MUFA. The G allele is also associated to higher *ACSL4* mRNA expression levels in liver than the A allele [15]. These SNPs may be used in breeding schemes with the purpose of improving meat quality by favoring a better fatty acid profile in terms of nutritional, organoleptic, technological and manufacturing properties. In the case of *SCD* mutation, the favorable allele is already at high frequencies in most populations, in agreement with the high desaturation potential and favorable fatty acid profile and meat quality parameters observed in these breeds [74,94–101]but there are few as Black Slavonian and Mora Romagnola with selection possibilities. For *ACSL4*, in contrast, high level of segregation is observed in most breeds.

Boar taint is an unpleasant odor that influences the smell and taste of cooked pork from non-castrated male pigs. This defect leads to losses in carcass value resulting in economic cost to the industry. Its main cause is the accumulation of androstenone and skatole in fat tissues. Alternatives to surgical castration for the control of this problem include selective breeding and in consequence many studies have addressed the genetic variation underlying androstenone and skatole levels. Many QTLs have been found [102] and a few candidate genes have been identified. Our panel included two genes involved in the levels of androstenone and skatole in porcine tissues. The cytochrome P450 II E1 (*CYP2E1*) gene encodes an enzyme involved in the degradation of skatole in the liver, being negatively correlated with its accumulation; the g.2412C>T (g. 141690107C>T in Sscrofa11.1) polymorphism located in the promoter of *CYP2E1* gene accounts for more than a 10% of total phenotypic variance of skatole content in backfat, the C allele being associated to higher skatole levels [22,23]. This unfavourable allele has high frequency in several of the breeds included in the current study, but intermediate or low in others such as Gascon, Moravka or Turopolje (≤0.36). The cytochrome b5 (*CYB5A*) gene codify for a protein involved in androstenone biosynthesis. A c.-8T>G polymorphism was found located 8bp upstream the start codon [20] and c.-8T allele was associated with low activity of CYB5A protein and low fat androstenone levels in different pig populations [20,21]. According to our results, the favorable c.-8T allele is scarce, with very low frequency in most of the examined local breeds (0 to 0.31). Only Iberian and Alentejana breeds showed intermediate frequencies for this polymorphism (≈ 0.5). Thus, in general, boar taint genes offer a wide margin for genetic selection and improvement in most of our breeds. This possibility is especially interesting in local breeds in which high fatness is characteristic and slaughter weights are usually very high, as body fat is positively associated with boar taint [103] and boar taint increases with body weight at slaughter [104].

Proteolytic enzymes participating in *postmortem* proteolysis are determinant factors in meat tenderization thus affecting eating quality. The calpastatin (*CAST*) gene codes for an inhibitor of proteases whose activity is highly related to meat tenderness in different species. More than 900 polymorphisms have been identified in this gene [19], the SNP g.49223G>A (g.103299934G>A, in Sscrofa11.1) here analyzed has been associated to tenderness and juiciness, being G the favorable allele [105]. Calpains are intracellular cysteine proteases also influencing *post-mortem* meat processes. Among them the calpain S1 (*CAPNS1*) polymorphism c.429A>C has been associated with pH, conductivity and meat color in different experimental and commercial pig populations [18]. Allele C was associated to higher pH, lower conductivity, lower cooking loss and lower lightness, thus reflecting better meat quality. Both SNPs showed a high level of segregation in local breeds, allowing the performance of association studies in all but Krškopolje and Turopolje breeds for *CAST* and *CAPNS1* genes, respectively, in which the favorable alleles are already at high frequency or even fixed.

Some genes involved in fat metabolism and deposition may also influence meat quality parameters. The peroxisome proliferator activated receptor gamma coactivator 1A (*PPARGC1A*) gene codes for a transcription coactivator involved in adipogenesis, energy metabolism and muscle fiber determination, favoring oxidative type fibers. The *PPARGC1A* c.1288T>A polymorphism was associated with pH and cooking loss in Duroc×Pietrain experimental cross and in Italian Large White and Landrace [18] showing additive and dominant effects. For this polymorphism segregation was observed in all breeds but Alentejana, Cinta Senese, Iberian and Turopolje. The adiponectin (*ADIPOQ*) gene encodes an adipokine involved in glucose and lipid metabolism. The non-synonymous polymorphism c.1735G>A had been previously associated with fat deposition [106] and was recently shown to have significant additive and dominant effects on several sensory traits such as flavor, tenderness, juiciness and meat color, with genotype AA being the most favorable [16]. In the investigated breeds, A allele is scarce which is in agreement with previous results in commercial crossbred pigs (frequency of A allele = 0.14) and would allow the implementation of selection procedures, or even absent in some ones, such as Apulo-Calabrese, Casertana, Cinta Senese, Gascon, Swallow-Bellied Mangalitsa and Turopolje.

Glycogen content is determinant of multiple meat quality traits, such as pH, meat color, drip loss or tenderness. One of the first genes identified affecting these meat quality traits in pigs was *PRKAG3* [48]. The protein encoded by this gene is a regulatory subunit of the AMP-activated protein kinase (AMPK) which plays a key role in the regulation of glucose and energy metabolism in skeletal muscle. Two main missense mutations have been widely studied in this gene. The *PRKAG3* p.R200Q mutation appears in Hampshire breed or derived synthetic lines. The mutated dominant allele leads to high glycogen deposition in muscle, high glycolytic potential and lactate concentration, decreasing meat quality (low pH, pale meat and low yield). This mutant A allele was absent in all local porcine breeds, in agreement with the main phenotypic characteristics of our local breeds and with previous works [107]. The p.V199I mutation, also involved in several meat quality traits, had been previously shown to segregate in different breeds, with allele A being most abundant in Iberian, Celta or Bísara, and less in most breeds selected for muscularity such as Duroc, Landrace and Pietrain [108]. The A allele, coding for isoleucine, leads to lower glycogen content, being favorable for meat quality [48]. According to our results this allele has a high frequency in Alentejana, Bísara, Casertana, Iberian, Swallow-Bellied Mangalitsa and Turopolje, in agreement with previous evidences.

More recently, a splice mutation in the phosphorylase kinase catalytic subunit gamma 1 (*PHKG1*) gene has been shown to be a causal mutation leading to high glycogen content and low meat quality in pig muscle [46]. This gene was studied as a positional candidate for a QTL located in SSC3 affecting glycolytic potential. The point mutation found produces a 32 bp deletion in the ORF and induces a premature stop codon; nonsense mediated decay of the transcript and ultimately lowers protein content and activity. The mutant A allele causes a 43% increase of glycolytic potential and a >20% decrease of water-holding capacity, with consistent negative effects on meat quality in different genetic backgrounds with Duroc origin [46]. According to our results, the favorable allele is predominant, either fixed or almost fixed in most breeds although in few of them (Apulo-Calabrese, Krškopolje, Lithuanian indigenous wattle, Moravka, and Sarda) the unfavorable A allele is at a relevant frequency ranging from 0.32 to 0.56.

The phosphoenolpiruvate carboxykinase (*PCK1*) gene codes for a regulatory molecule involved in gluconeogenesis. A polymorphism in this gene (c.2456A>C, p.M139L) has been recently reported [45] as causal mutation associated to intramuscular fat content, backfat thickness and meat quality in pigs. The c.2456C allele encoding leucine has negative effects on these traits, and is present in many breeds or crosses, especially in the ones selected for high lean content, and even in wild pigs although at substantial different frequencies. The *PCK1* c.2456A allele, the one that encodes methionine and is associated to both less water losses and more favorable fat distribution, seems to be overrepresented in breeds or crosses not subjected to strong artificial selection [45]. Our results show that, in fact, a few traditional non-selected breeds, such as Iberian and Alentejana, have the favorable allele at a very high frequency, or even fixed in the case of Swallow-Bellied Mangalitsa. Conversely, the rest of analysed breeds show small (Gascon, Lithuanian indigenous wattle and Turopolje) or intermediate frequencies. Despite the fatness of Lithuanian indigenous wattle pigs their intramuscular fat content is quite low. In these breeds, selection of this PCK1 c.2456A allele could favor the desirable phenotype in pig breeding schemes, with increased intramuscular fat content, better meat quality and reduced amounts of subcutaneous and visceral fat.

### Markers related to disease resistance

Three genes involved in disease resistance traits were explored (Table 5). The guanylate binding protein 5 (*GBP5*) gene is known to play a role in host immune response and inflammation based on studies in the knockout mouse. The rs340943904 mutation in the pig gene is a strong candidate causal mutation for a QTL on SSC4 that controls variation in host response to Porcine Respiratory and Reproductive Syndrome (PRRS) virus [29]. This intronic mutation introduces a splice acceptor site which leads to a shifted reading frame and early stop codon that truncates the 88 C-terminal amino acids of the protein. The unfavorable G allele codes for the truncated GBP5 protein which is not able to inhibit viral entry and replication as quickly as the intact GBP5 protein [28] thus leading to a poor outcome of homozygous individuals following PRRS virus infection. Moreover, the quoted SNP has been shown to be a cis eQTL [30]. With the exception of three breeds showing intermediate frequencies (Cinta Senese, Lithuanian indigenous wattle and Mora Romagnola), the unfavorable allele shows very high frequency in all breeds (>0.73), opening potential selection possibilities. Marker assisted selection to increase the frequency of T allele, corresponding to increased PRRS resistance, has been proposed to reduce PRRS viral load, thus decreasing the costs associated with PRRS by reducing its incidence, because a lower viral burden may also reduce virus shedding [109]. Besides, such selection would be expected to improve weight gain under infection. However, the amount of response is limited because the SNP explains only a portion of the genetic variance in host response. Thus, genomic selection for viral load, in combination with marker-assisted selection on the GBP5 genotype, may hold potential for improved resistance to PRRS in some of the breeds.

Enterotoxigenic *Escherichia coli* (ETEC), that expresses the F4ab and F4ac fimbriae is a major cause of diarrhea outbreaks and mortality in the pig breeding industry, infecting both newborn and weaned piglets. The mucin 4 gene (*MUC4*) SNP located in intron 7 (DQ848681:g.8227C>G, g. 134226654C>G in Sscrofa11.1) is the most extensively studied polymorphism in relation to ETEC F4ab/ac susceptibility in pigs [43]. This SNP is also associated to growth performance, with significant effects on average daily gain and backfat thickness [110]. The C allele, associated with susceptibility, dominates the resistant G allele [111]. According to this work the polymorphism is in complete linkage disequilibrium with the susceptibility phenotype and is currently used as a genetic test in the Danish pig breeding industry. Our results indicate that the unfavorable allele is majoritarian or even fixed in most populations, with the exception of both Lithuanian and Bísara breeds in which intermediate frequencies (≤0.60) are observed.

Weaned piglets are also susceptible to F18-positive Escherichia coli (ETECF18) infections leading to post-weaning diarrhea or edema disease. The F18 receptor (F18R) plays a crucial role in this disease by mediating the binding of F18 fimbriated bacteria to the intestinal epithelium with F18R positive animals being susceptible to infection. Susceptibility to this infection appears to be dependent on the activity of the alpha-fucosyltransferase-1 (*FUT1*) gene, considered the candidate gene controlling the adhesion to F18 receptor. In this gene a G/A transition resulting in an p.A103T amino acid substitution has been discovered which is involved in susceptibility, AA individuals are resistant to ETECF18 while GG or AG are sensitive. This mutation of *FUT1* gene has been also associated to resistance to natural infection by PRRS and *Haemophilus parasuis* virus [27], thus it might play a role in pig infection by multi-pathogens, with AA being a favorable genotype for increasing the resistance to disease. The favorable A allele has also been associated to a better growth and development, better meat quality, lower fat content and higher fetal survival [112] and has been shown to be scarce in many pig breeds [27,113]. In most of our breeds, G allele is present at high or intermediate frequency, with only Krškopolje and Turopolje showing high frequency of the favorable allele A. Marker assisted selection to increase the frequency of the A allele not only would enhance the anti-disease ability, but would also improve the growth, meat quality, carcass traits and reproductive ability.

### Markers associated with prolificacy

The estrogen receptor 1 (*ESR1*) gene polymorphism, previously associated to litter size [24], was not polymorphic in any of the analyzed breeds. The allele responsible for a higher prolificacy, with Chinese origin and predominant in Meishan and also found in Large White [114], is absent in our local breeds, with the unfavorable allele in terms of litter size being fixed in all of them (Table 5). This result is in agreement with previously published reports in some of the analyzed breeds, such as Swallow-Bellied Mangalitsa [49] and with the general low reproductive ability of autochthonous breeds. Results suggest lack or low influence from Chinese pig breeds in those tested in this work.

The aryl hydrocarbon receptor (*AHR*) gene polymorphism shows segregation at intermediate frequencies in most of tested populations. Iberian and Alentejana breeds showed the wild allele almost fixed. For this Asian-derived non-synonymous mutation the mutant G allele has been associated with a substantial increase in litter size in multiple European commercial lines [17]. The gene has also been related to fertility and embryo development in other mammals [115,116]. Again, the genotyping results agree with the characteristic low prolificacy observed in local unselected breeds.

### Genetic diversity in candidate genes

The results of diversity parameters obtained for each tested and segregating marker are shown in Table 6. The observed heterozygosity (H_O_) and expected heterozygosity (H_S_) values per locus ranged from 0.024 to 0.414 and from 0.025 to 0.415, respectively, with overall values of 0.24 for both parameters. F_ST_ is a measure of population structure due to genetic structure and its values range from 0 to 1. While a zero value indicates there is no genetic structures and the populations are in complete panmixia, a value of one implies there are not gene flux between the populations. The overall F_ST_ value from all tested loci was 0.25, pointing out that a 25% of genetic differences are due to differences between breeds and a 75% is caused by differences among individuals, therefore, the breeds studied share some genetic diversity. A similar value (F_ST_ = 0.27) [117] was also observed in an study including 11 European breeds (Pietrain, Sortbroget, Basque, Gascon, Limousin, Normand, German Landrace, Schwäbisch-Hällisches, Great Yorkshire, Swedish Landrace, European Wild Pig). Highest F_ST_ values were obtained for *MC1R* and *KIT* loci, meaning that these markers show the highest degree of differentiation among populations, whereas the lowest values were observed for *FASN*, *PPARD* and *RYR1* polymorphisms.

**Table 6:** Observed (Ho) and expected heterozygosity (H_S_) and F_ST_ values by locus, averaged among all the breeds.

Gene	H_O_	H_S_	F_ST_
*ACACA*	0.424	0.425	0.122
*ACSL4*	0.198	0.331	0.260
*ADIPOQ*	0.113	0.115	0.137
*AHR*	0.379	0.394	0.158
*CAPNS1*	0.378	0.382	0.221
*CAST*	0.295	0.389	0.206
*CYB5A*	0.195	0.194	0.177
*CYP2E1*	0.331	0.329	0.232
*FASN*	0.660	0.471	0.053
*FTO*	0.371	0.382	0.220
*FUT1*	0.294	0.302	0.231
*GBP5*	0.237	0.241	0.159
*IGF2*	0.185	0.200	0.298
*KIT*	0.099	0.094	0.591
*LEP*	0.364	0.365	0.270
*LEPR*	0.223	0.274	0.384
*MC1R*	0.280	0.265	0.601
*MC4R*	0.321	0.306	0.269
*MSTN*	0.354	0.352	0.253
*MTTP*	0.414	0.415	0.150
*MUC4*	0.282	0.269	0.112
*NR6A1*	0.132	0.129	0.201
*PCK1*	0.330	0.343	0.304
*PHKG1*	0.052	0.150	0.313
*PPARD*	0.074	0.079	0.065
*PPARGC1A*	0.378	0.375	0.248
*PRKAG3 I199V*	0.399	0.388	0.214
*RYR1*	0.048	0.053	0.076
*SCD*	0.228	0.218	0.269
*TAS2R39*	0.167	0.172	0.129
*TYRP1*	0.024	0.025	0.103

The global Ho, Hs and F_IS_ values for each breed are shown in Table 7. The highest Ho and Hs values are observed in Moravka, Krškopolje and Sarda indicating a higher degree of genetic diversity than other breeds such as Basque, Alentejana and Iberian breeds, which exhibit the lowest values. F_IS_ estimates the departure from panmixia at the subpopulation level, and is a measure of inbreeding; negative values indicate there are less related individuals than expected by random mating and positive values indicate there are more related individuals than expected by random mating. According to this statistic, Casertana and Apulo Calabrese are the breeds displaying the highest inbreeding, in agreement with their endangered situation and small census [99,118,119].

**Table 7:** Diversity parameters per breed.

Breed	H_O_	H_S_	F_IS_
Alentejana	0.155	0.170	0.062
Apulo Calabrese	0.223	0.260	0.115
Basque	0.153	0.152	0.024
Bísara	0.259	0.274	0.065
Black Slavonian	0.288	0.271	-0.061
Casertana	0.216	0.239	0.131
Cinta Senese	0.247	0.249	0.016
Gascon	0.224	0.214	-0.003
Iberian	0.165	0.185	0.084
Krškopolje	0.310	0.302	-0.029
Lithuanian indigenous wattle	0.281	0.288	0.009
Lithuanian White Old Type	0.237	0.233	0.022
Majorcan Black	0.232	0.238	0.041
Mora Romagnola	0.195	0.187	-0.001
Moravka	0.310	0.315	0.007
Nero Siciliano	0.260	0.281	0.074
Sarda	0.297	0.325	0.085
Schwäbisch-Hällisches Schwein	0.250	0.250	0.006
Swallow-Bellied Mangalitsa	0.191	0.197	0.050
Turopolje	0.209	0.186	-0.096

**Fig 2:**
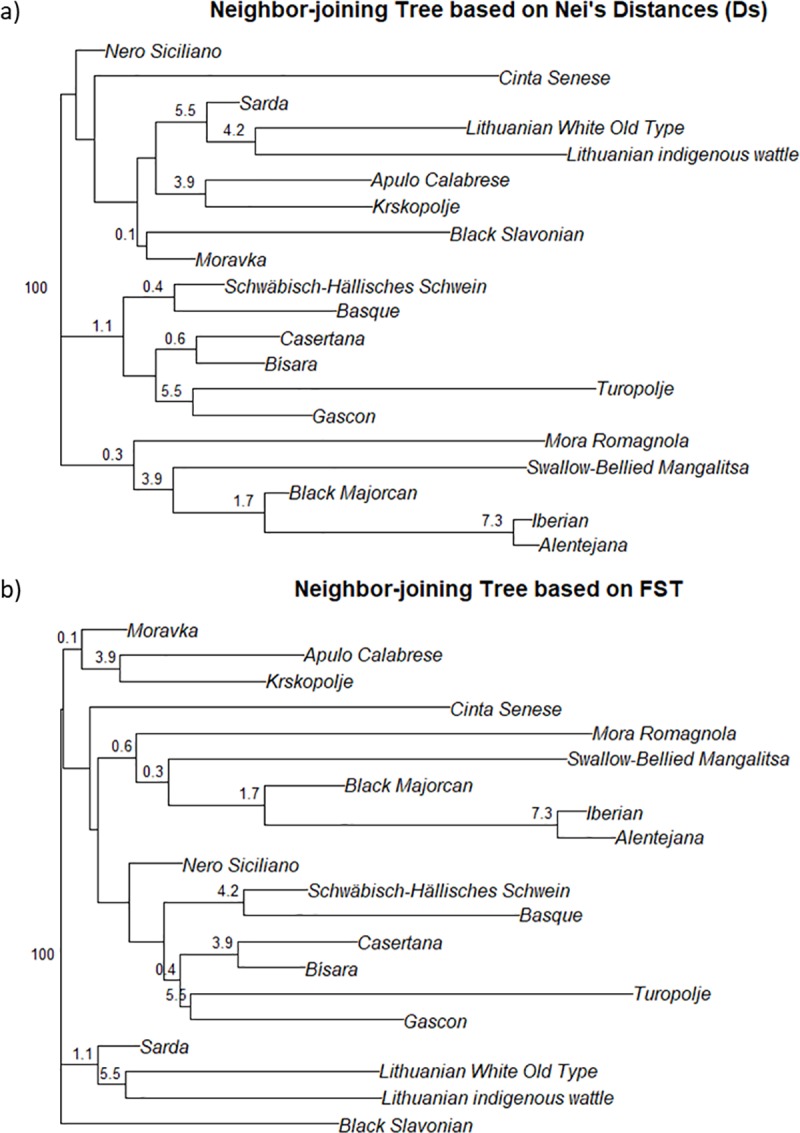
Neighbour-joining tree based on a) Nei’s distances b) F_ST_.

Nei’s genetic distances ranged from 0.007 between Alentejana and Iberian breeds to 0.240 between Black Slavonian and Turopolje. The F_ST_ pairwise estimates ranged from 0.039 between Alentejana and Iberian breeds to 0.524 between Mora Romagnola and Turopolje (S2 and S3 Tables). Fig 2A and 2B show the phylogenetic trees constructed using these distances, bootstrapping values were very low, which point out there is not enough information to solve the phylogenetic trees. However, both trees agree at least partially with geographic distribution of breeds, with those that are geographically close grouping into the same branches, as Alentejana, Iberian and Majorcan Black and the two Lithuanian breeds. Regarding the breeds coming from the Iberian Peninsula, the Portuguese Bisara breed is, according to SNP genotyping, apparently unrelated to the rest. Herrero-Medrano et al. [120] observed this separation between Iberian and Bisara breeds, which agrees with their different Iberian and Celtic origins [121].

### Genetic structure of populations using DAPC and STRUCTURE

**Fig 3:**
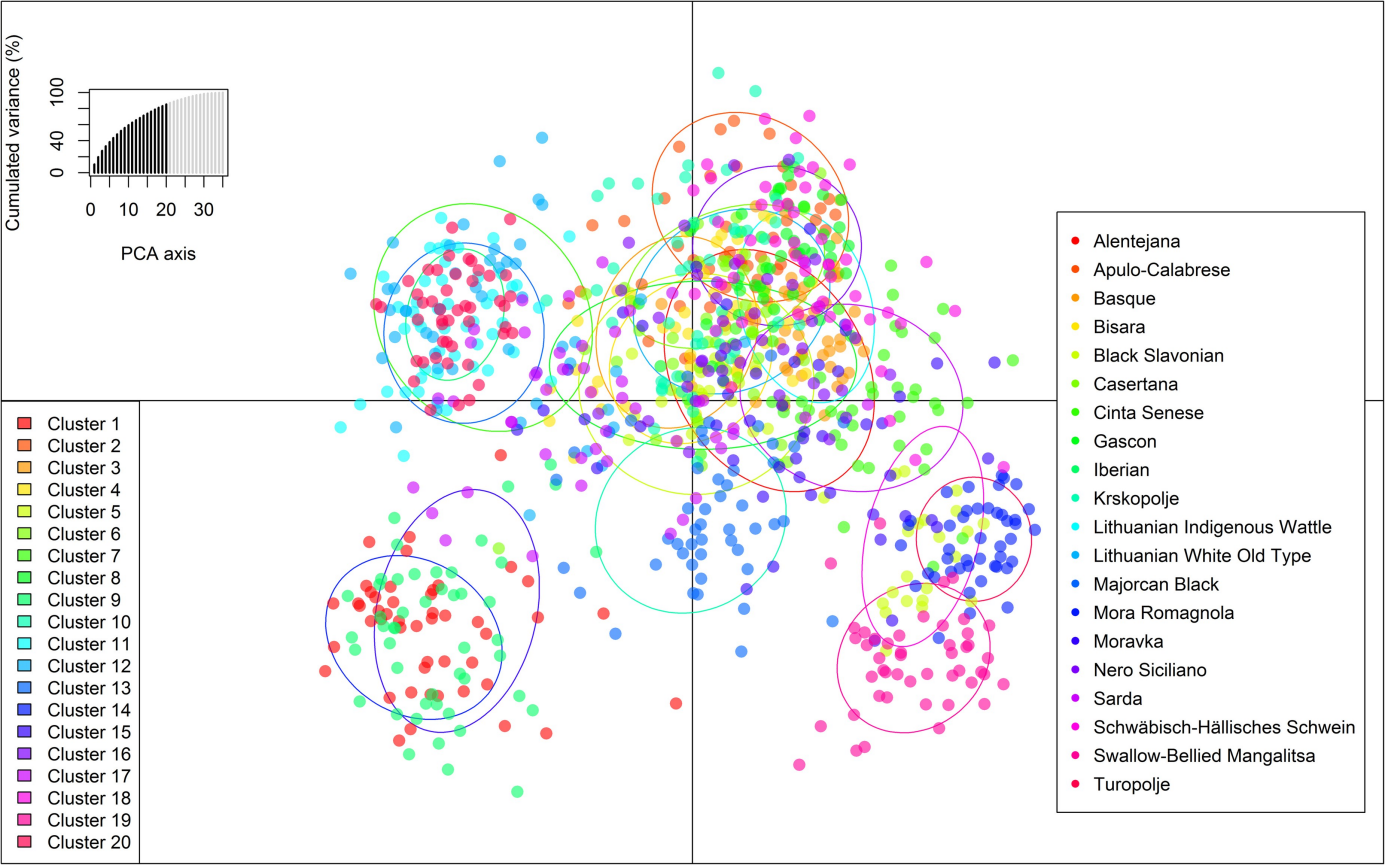
Scatterplot of resulting genetic clusters after Discriminant Analysis of Principal Components for the 20 local European pig breeds.

Based on the lowest BIC value found (S1 Fig) a total of 20 clusters were detected, which correspond to the number of the original local European pig breeds considered in the analysis. DAPC analysis was thus performed retaining 20 PCs, which explain more than 90% of the total variance, and seven discriminant eigenvalues. The resulting representation of the first two Linear Discriminants (Fig 3) was not able to individualize clusters formed by individuals belonging to a specific breed but it is also true that variance explained by these first two components represented only 20% of total variance. Both α-score optimization (S2 Fig) and cross validation (S3 Fig) procedures identify 19 PCs to be retained, which is quite close to the value of 20 found with DAPC. Actually, the lowest RMSE for CV corresponds to 30 PCs but it was only slightly lower than the value found for 19 PCs (0.181 vs 0.191), both obtaining a mean successful assignment greater than 80%. Furthermore, the scatterplot of the DAPC cross-validation is rather flat after 15 retained PCs and thus it is not advisable retaining too many PC axes in order to not create an excessively complex model, not suitable to analyze unseen data.

**Fig 4:**
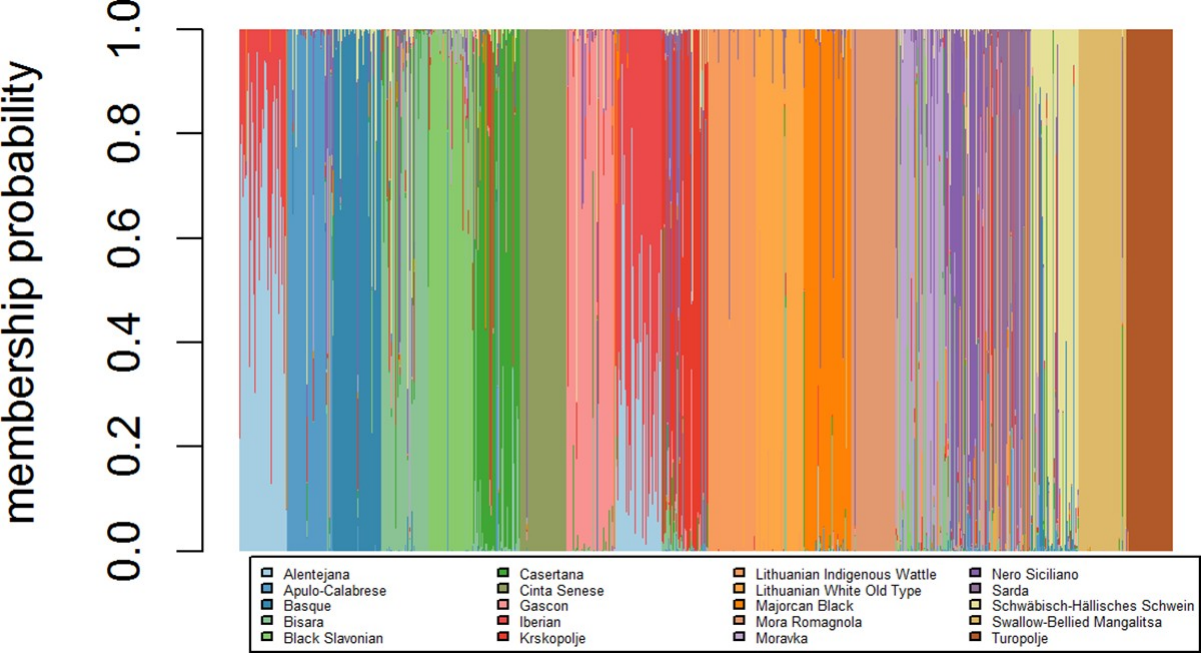
Membership assignment of the individuals to the *a priori* clusters defined with DAPC.

Group membership based on the retained discriminant functions (Fig 4) showed a rather clear distinction among the different populations with high genetic structure for Mora Romagnola, Turopolje and Swallow-Bellied Mangalitsa pig breeds, followed by Cinta Senese, Gascon and Lithuanian indigenous wattle breeds which presented few admixed individuals within their respective clusters. For most of the other breeds, a general framework of admixed history is evident, even if no clear pattern is recognizable. On the contrary, Alentejana and Iberian breeds were basically included in two shared clusters separated from the other populations confirming the common past history of the two breeds. The proportion of successful reassignment using the 19 Linear Discriminant functions suggested by DAPC is showed in S4 Fig. Some breeds are almost fully reassigned to the original population (i.e. Turopolje, Lithuanian indigenous wattle, Mora Romagnola, Swallow-Bellied Mangalitsa, and Cinta Senese) whereas the lowest proportion of reassignment has been registered for Sarda, Nero Siciliano and Moravka breeds. Indeed, the breeds with a high proportion of successful reassignment are those with a clearly distinct pattern of membership assignment whereas the strongly admixed populations are those with values of correct reassignment below 80%. The same reassignments has been performed using randomized groups of individuals in or-der to be protected against the extraction of residual variation considered as explanatory of the underlying model structure (i.e. overfitting) (S5 Fig) and the results are quite comfortable; the highest successful proportion of reassignment was slightly higher than 30%, thus confirming the reliability of the discrimination process.

The genetic structure of the different populations was also studied through a Bayesian clustering method that requires high computational time and which accuracy strictly depends on the underlying population genetic model behind the original data [122]. The combination of the modal value of the ΔK (S6A Fig) and the mean variance (S6B Fig) of the ln Pr(G|K) is used as an indicator of the strength of the signal detected by the software. Our results showed the strongest signal at k = 14 with another significant peak at k = 21 with mean and standard deviation of LnP(K) of -23894.17 and -23124.02, and 36.02 and 37.30 for k = 14 and k = 21, respectively.

**Fig 5:**
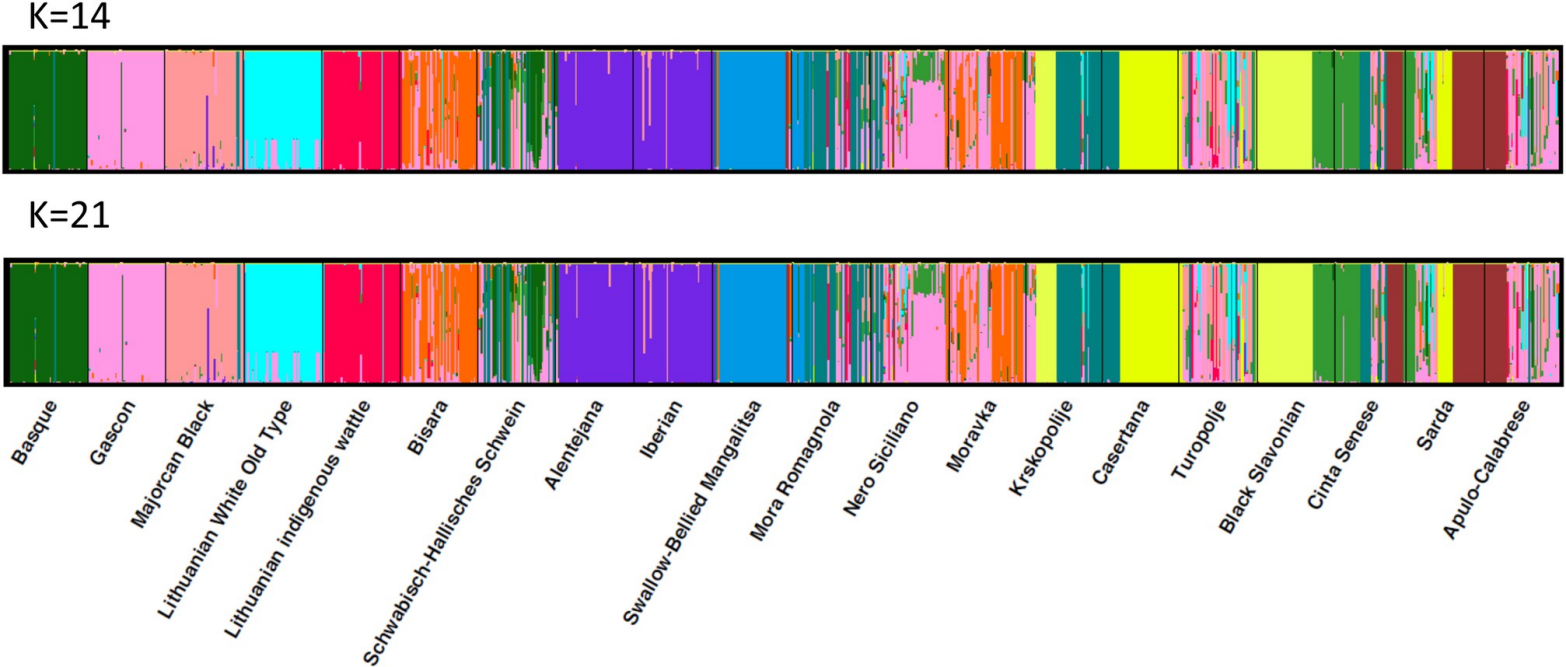
Genetic diversity structure of the 20 pig populations. Population memberships for each genotype is shown based on K = 14 and K = 21.

The level of admixture was uneven in the different populations when looking at the individual membership (Fig 5). As already stated with DAPC, Alentejana and Iberian breeds clustered together both at k = 14 and k = 21 but in general the software was able to better assign the individuals to specific clusters. The highest numbers of admixed individuals were detected for Bísara, Schwäbisch-Hällisches Schwein, Mora Romagnola, Nero Siciliano, Moravka and Turopoljie breeds but also some other breeds (i.e. Cinta Senese, Sarda, and Apulo-Calabrese breeds) showed a certain degree of admixture among the individuals. The increase of k from 14 to 21 allows separating some breeds formerly clustered with other populations (f.i. Crna Slavonska and Casertana breeds) but it was not conclusive in terms of assignment, confirming the admixed history of the breeds. These results are not fully superimposable to those found with DAPC analysis, suggesting that the Bayesian clustering method was able to find hidden structures of the data, not easily recognizable with the previous one.

Results provide insights into the different population’s genetic structure, relationships and clustering among them. Nevertheless, the genotyping data employed seems not sufficient to clarify the complex relationships in this large collection of phenotypically close pig breeds. The use of larger panels of neutral marker loci is probably advised in order to deepen in their genetic structure and to relate it with their breeding history.

### Final considerations

We report a comprehensive analysis of genetic variation and population genetic structure based on a panel of candidate genes in a wide panel of European local pig breeds. Results are in agreement with known facts of the breeds’ origin and their phenotype. A clear genetic differentiation and intra-breed homogeneity was observed in some cases, while other breeds showed a high degree of admixture. Alleles with contrasted effects on production and fatness (such as *LEPR*, *FTO*, *MC4R*, *LEP* or *MSTN*), meat quality (*PCK1*, *PRKAG3*, *ACACA*, *CAST*, *MTT*P) or disease resistance (*MUC4*, *GBP5*) are segregating in many breeds, in some cases with intermediate frequencies, opening selection possibilities. Results provide an extended catalogue of fixed and segregating candidate SNPs associated with complex traits with potential usefulness for traceability purposes and for association studies and breeding programs. For a practical application, the percentage of variance of the traits of interest explained by the polymorphisms must now be further studied in each breed. If sufficient to justify the genotyping cost, a marker assisted selection could be implemented.

## Supporting information

S1 Table Genotyping data. (XLSX)

S2 Table Nei’s genetic distances computed between each pair of pig populations. (DOCX)

S3 Table FST computed between each pair of pig populations. (DOCX)

S1 Fig Graph of BIC value vs. number of detected clusters. (TIF)

S2 Fig Graph of α-score optimization of DAPC. (TIF)

S3 Fig Graph of cross-validation of DAPC. x axis reports the number of PC retained in each DAPC and y axis the proportion of successful outcome prediction.
(TIF)

S4 Fig Proportion of successful reassignment of the individuals to their specific breed. (TIFF)

S5 Fig Proportion of successful reassignment using randomized groups of individuals. (TIFF)

S6 Fig The most likely number of clusters (K) in the analyzed dataset. a) plot of ln Pr(G|K) values as a function of the number of clusters for each of the 20 runs carried out for each K value using the program STRUCTURE. b) plot of K as the mean of the absolute values of L”(K) averaged over 20 runs divided by the standard deviation of L(K) between successive K values.
(TIF)
