# Supplementary material for: Diversity across major and candidate genes in European local pig breeds
Source: PLoS One. 2018 Nov 20;13(11):e0207475. doi: 10.1371/journal.pone.0207475 (PMC6245784; doi:10.1371/journal.pone.0207475)
Supplement: S2 Table — (DOCX) [file pone.0207475.s002.docx]

Supplementary Table 2. Nei’s genetic distances computed between each pair of pig populations.

|  | AL | AP | BA | BI | BS | CA | CS | GA | IB | KR | LI | LW | MB | MR | MO | NS | SA | SC | SW |
| --- | --- | --- | --- | --- | --- | --- | --- | --- | --- | --- | --- | --- | --- | --- | --- | --- | --- | --- | --- |
| AP | 0.152 |  |  |  |  |  |  |  |  |  |  |  |  |  |  |  |  |  |  |
| BA | 0.128 | 0.084 |  |  |  |  |  |  |  |  |  |  |  |  |  |  |  |  |  |
| BI | 0.118 | 0.086 | 0.063 |  |  |  |  |  |  |  |  |  |  |  |  |  |  |  |  |
| BS | 0.207 | 0.155 | 0.161 | 0.156 |  |  |  |  |  |  |  |  |  |  |  |  |  |  |  |
| CA | 0.130 | 0.083 | 0.068 | 0.026 | 0.164 |  |  |  |  |  |  |  |  |  |  |  |  |  |  |
| CS | 0.179 | 0.109 | 0.113 | 0.130 | 0.196 | 0.122 |  |  |  |  |  |  |  |  |  |  |  |  |  |
| GA | 0.153 | 0.101 | 0.068 | 0.050 | 0.197 | 0.051 | 0.094 |  |  |  |  |  |  |  |  |  |  |  |  |
| IB | 0.007 | 0.150 | 0.142 | 0.116 | 0.217 | 0.123 | 0.170 | 0.152 |  |  |  |  |  |  |  |  |  |  |  |
| KR | 0.171 | 0.055 | 0.101 | 0.053 | 0.164 | 0.063 | 0.116 | 0.049 | 0.165 |  |  |  |  |  |  |  |  |  |  |
| LI | 0.151 | 0.093 | 0.148 | 0.092 | 0.196 | 0.103 | 0.147 | 0.129 | 0.134 | 0.098 |  |  |  |  |  |  |  |  |  |
| LW | 0.131 | 0.096 | 0.137 | 0.087 | 0.175 | 0.106 | 0.130 | 0.120 | 0.129 | 0.080 | 0.088 |  |  |  |  |  |  |  |  |
| MB | 0.057 | 0.083 | 0.078 | 0.061 | 0.155 | 0.065 | 0.116 | 0.090 | 0.057 | 0.084 | 0.147 | 0.120 |  |  |  |  |  |  |  |
| MR | 0.185 | 0.179 | 0.124 | 0.123 | 0.185 | 0.122 | 0.169 | 0.124 | 0.169 | 0.186 | 0.213 | 0.220 | 0.132 |  |  |  |  |  |  |
| MO | 0.110 | 0.050 | 0.083 | 0.064 | 0.082 | 0.072 | 0.092 | 0.072 | 0.112 | 0.055 | 0.109 | 0.086 | 0.062 | 0.125 |  |  |  |  |  |
| NS | 0.106 | 0.049 | 0.041 | 0.044 | 0.128 | 0.053 | 0.073 | 0.036 | 0.105 | 0.051 | 0.091 | 0.075 | 0.047 | 0.099 | 0.037 |  |  |  |  |
| SA | 0.079 | 0.056 | 0.079 | 0.052 | 0.155 | 0.058 | 0.083 | 0.069 | 0.071 | 0.046 | 0.059 | 0.057 | 0.048 | 0.140 | 0.045 | 0.040 |  |  |  |
| SC | 0.158 | 0.055 | 0.037 | 0.044 | 0.130 | 0.042 | 0.121 | 0.050 | 0.170 | 0.047 | 0.131 | 0.101 | 0.076 | 0.141 | 0.052 | 0.038 | 0.067 |  |  |
| SW | 0.140 | 0.143 | 0.106 | 0.116 | 0.152 | 0.107 | 0.130 | 0.164 | 0.146 | 0.165 | 0.192 | 0.201 | 0.078 | 0.123 | 0.088 | 0.109 | 0.110 | 0.113 |  |
| TU | 0.137 | 0.162 | 0.123 | 0.094 | 0.240 | 0.084 | 0.191 | 0.088 | 0.151 | 0.102 | 0.159 | 0.111 | 0.136 | 0.238 | 0.131 | 0.107 | 0.098 | 0.091 | 0.200 |

AL:Alentejana; AP: Apulo Calabrese; BA: Basque; BI: Bísara; BS: Black Slavonian; CA: Casertana; CS: Cinta Senese; GA: Gascon; IB: Iberian; KR: Krskopolje; LI: Lithuanian indigenous wattle; LW: Lithuanian White Old Type; MB: Majorcan Black; MR: Mora Romagnola; MO: Moravka; NS: Nero Siciliano; SA: Sarda; SC: Schwäbisch-Hällisches Schwein; SW: Swallow-Bellied Mangalitsa; TU: Turopolje
