# Supplementary material for: Diversity across major and candidate genes in European local pig breeds
Source: PLoS One. 2018 Nov 20;13(11):e0207475. doi: 10.1371/journal.pone.0207475 (PMC6245784; doi:10.1371/journal.pone.0207475)
Supplement: S3 Table — (DOCX) [file pone.0207475.s003.docx]

Supplementary Table 3. F_ST_ computed between each pair of pig populations.

|  | AL | AP | BA | BI | BS | CA | CS | GA | IB | KR | LI | LW | MB | MR | MO | NS | SA | SC | SW |
| --- | --- | --- | --- | --- | --- | --- | --- | --- | --- | --- | --- | --- | --- | --- | --- | --- | --- | --- | --- |
| AC | 0.381 |  |  |  |  |  |  |  |  |  |  |  |  |  |  |  |  |  |  |
| BA | 0.423 | 0.273 |  |  |  |  |  |  |  |  |  |  |  |  |  |  |  |  |  |
| BI | 0.321 | 0.216 | 0.217 |  |  |  |  |  |  |  |  |  |  |  |  |  |  |  |  |
| BS | 0.434 | 0.319 | 0.393 | 0.312 |  |  |  |  |  |  |  |  |  |  |  |  |  |  |  |
| CA | 0.362 | 0.224 | 0.244 | 0.082 | 0.341 |  |  |  |  |  |  |  |  |  |  |  |  |  |  |
| CS | 0.422 | 0.267 | 0.336 | 0.292 | 0.371 | 0.299 |  |  |  |  |  |  |  |  |  |  |  |  |  |
| GA | 0.414 | 0.271 | 0.260 | 0.156 | 0.395 | 0.168 | 0.261 |  |  |  |  |  |  |  |  |  |  |  |  |
| IB | 0.039 | 0.369 | 0.435 | 0.307 | 0.435 | 0.339 | 0.400 | 0.402 |  |  |  |  |  |  |  |  |  |  |  |
| KR | 0.381 | 0.144 | 0.288 | 0.135 | 0.308 | 0.167 | 0.259 | 0.145 | 0.364 |  |  |  |  |  |  |  |  |  |  |
| LI | 0.355 | 0.217 | 0.365 | 0.210 | 0.345 | 0.243 | 0.304 | 0.298 | 0.323 | 0.209 |  |  |  |  |  |  |  |  |  |
| LW | 0.367 | 0.253 | 0.391 | 0.228 | 0.358 | 0.282 | 0.314 | 0.321 | 0.354 | 0.204 | 0.221 |  |  |  |  |  |  |  |  |
| MB | 0.204 | 0.224 | 0.269 | 0.173 | 0.330 | 0.195 | 0.288 | 0.261 | 0.199 | 0.210 | 0.309 | 0.305 |  |  |  |  |  |  |  |
| MR | 0.478 | 0.407 | 0.404 | 0.319 | 0.399 | 0.337 | 0.399 | 0.357 | 0.447 | 0.390 | 0.419 | 0.471 | 0.354 |  |  |  |  |  |  |
| MO | 0.273 | 0.123 | 0.235 | 0.146 | 0.175 | 0.174 | 0.204 | 0.184 | 0.269 | 0.122 | 0.211 | 0.202 | 0.154 | 0.288 |  |  |  |  |  |
| NS | 0.292 | 0.135 | 0.154 | 0.118 | 0.268 | 0.150 | 0.188 | 0.116 | 0.283 | 0.128 | 0.204 | 0.201 | 0.135 | 0.271 | 0.089 |  |  |  |  |
| SA | 0.220 | 0.138 | 0.231 | 0.125 | 0.283 | 0.149 | 0.191 | 0.180 | 0.197 | 0.107 | 0.131 | 0.149 | 0.128 | 0.314 | 0.095 | 0.098 |  |  |  |
| SH | 0.387 | 0.154 | 0.148 | 0.123 | 0.281 | 0.128 | 0.283 | 0.158 | 0.393 | 0.125 | 0.275 | 0.259 | 0.207 | 0.352 | 0.126 | 0.106 | 0.157 |  |  |
| SB | 0.407 | 0.352 | 0.361 | 0.301 | 0.351 | 0.304 | 0.336 | 0.411 | 0.406 | 0.357 | 0.390 | 0.443 | 0.244 | 0.367 | 0.222 | 0.282 | 0.263 | 0.299 |  |
| TU | 0.412 | 0.387 | 0.402 | 0.267 | 0.457 | 0.264 | 0.427 | 0.286 | 0.422 | 0.270 | 0.358 | 0.323 | 0.361 | 0.524 | 0.298 | 0.286 | 0.250 | 0.267 | 0.477 |

AC: Apulo Calabrese; BA: Basque; BI: Bísara; BS: Black Slavonian; CA: Casertana; CS: Cinta Senese; GA: Gascon; IB: Iberian; KR: Krskopolje; LI: Lithuanian indigenous wattle; LW: Lithuanian White Old Type; MB: Majorcan Black; MR: Mora Romagnola;MO: Moravka; NS: Nero Siciliano; SA: Sarda; SH: Schwäbisch-Hällisches Schwein; SB: Swallow-Bellied Mangalitsa; TU: Turopolje
