# Supplementary figures and images for: Diversity across major and candidate genes in European local pig breeds
Source: PLoS One. 2018 Nov 20;13(11):e0207475. doi: 10.1371/journal.pone.0207475 (PMC6245784; doi:10.1371/journal.pone.0207475)

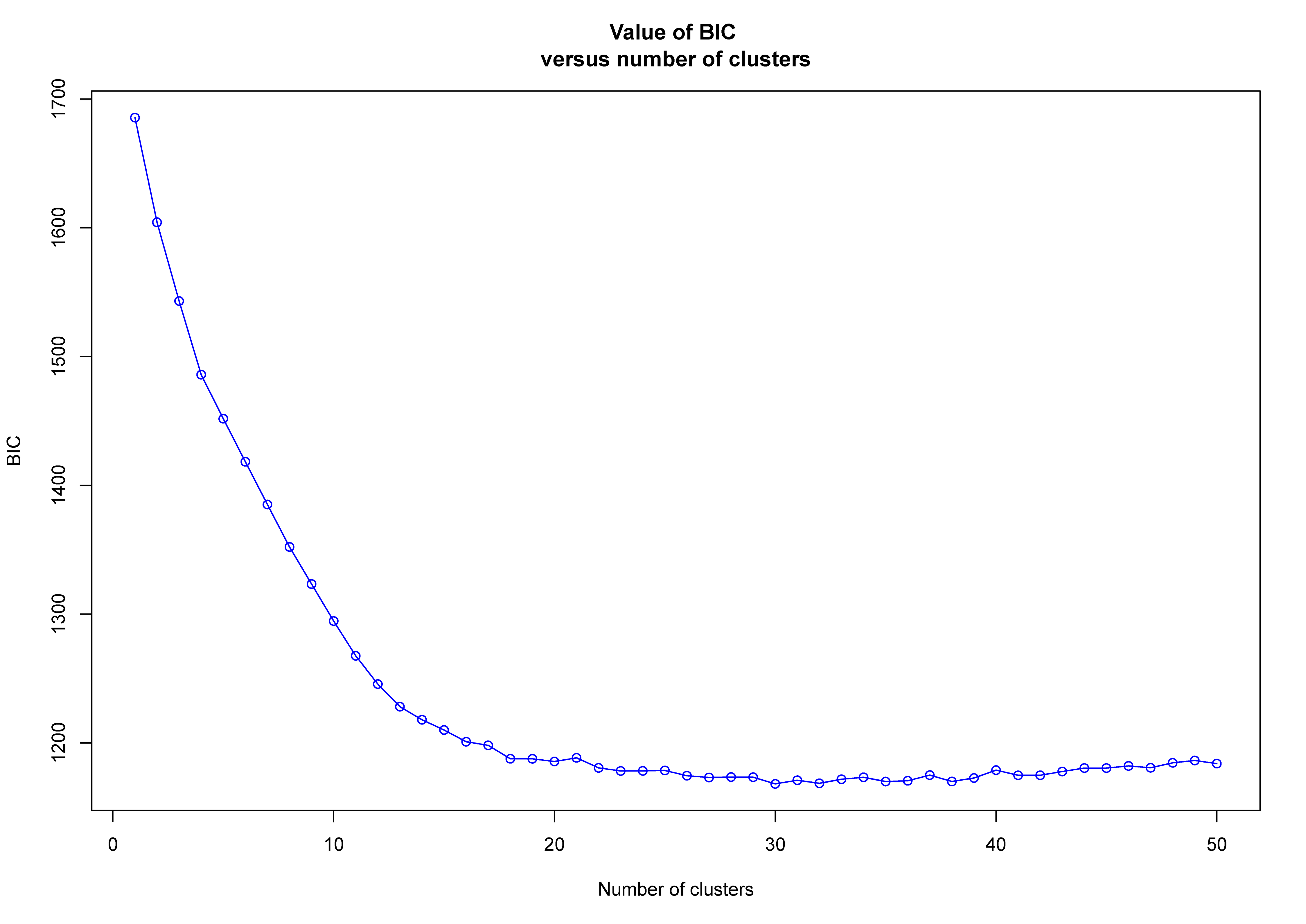

Supplement: S1 Fig — (TIF) [file pone.0207475.s004.tif]

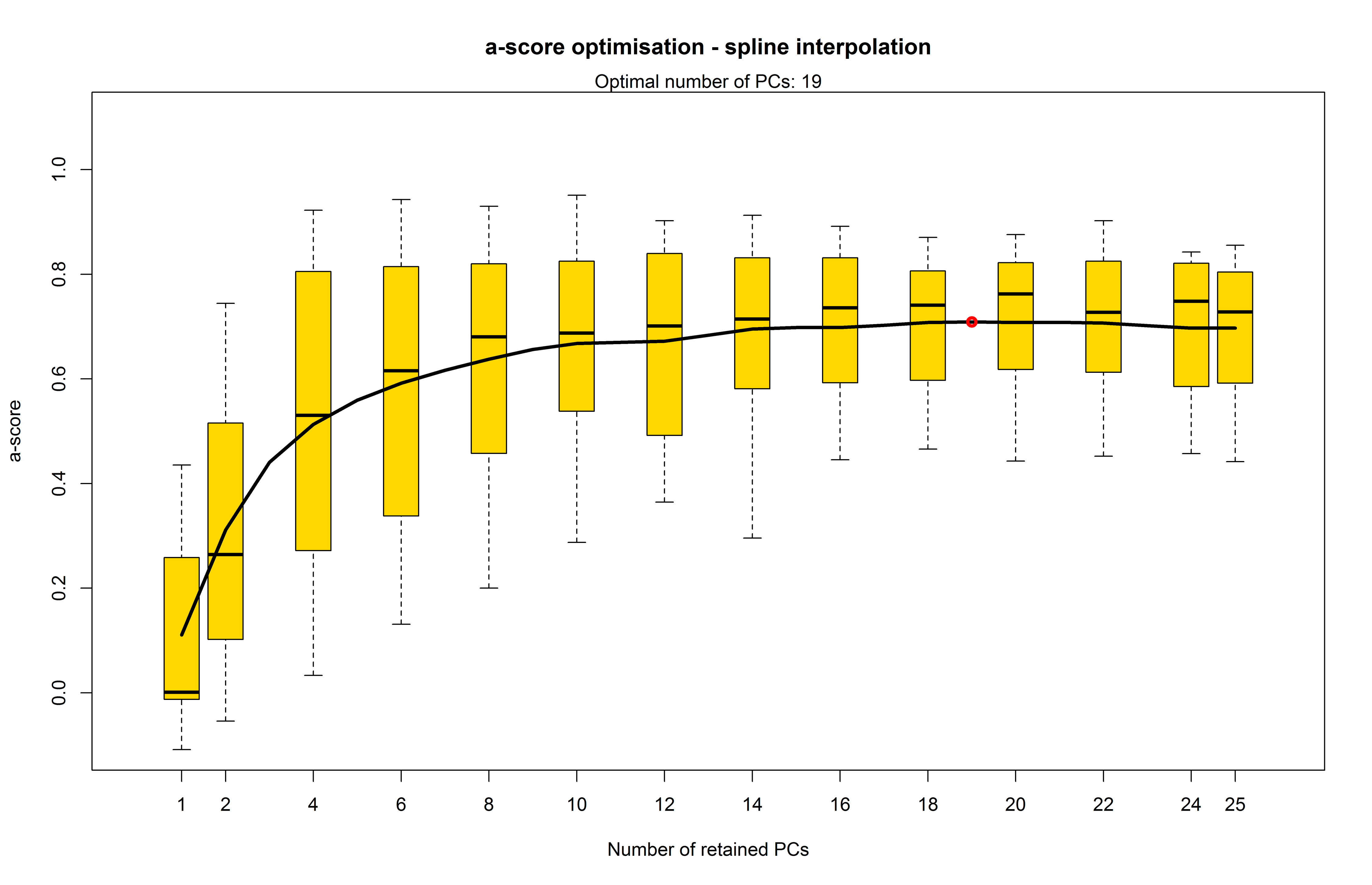

Supplement: S2 Fig — (TIF) [file pone.0207475.s005.tif]

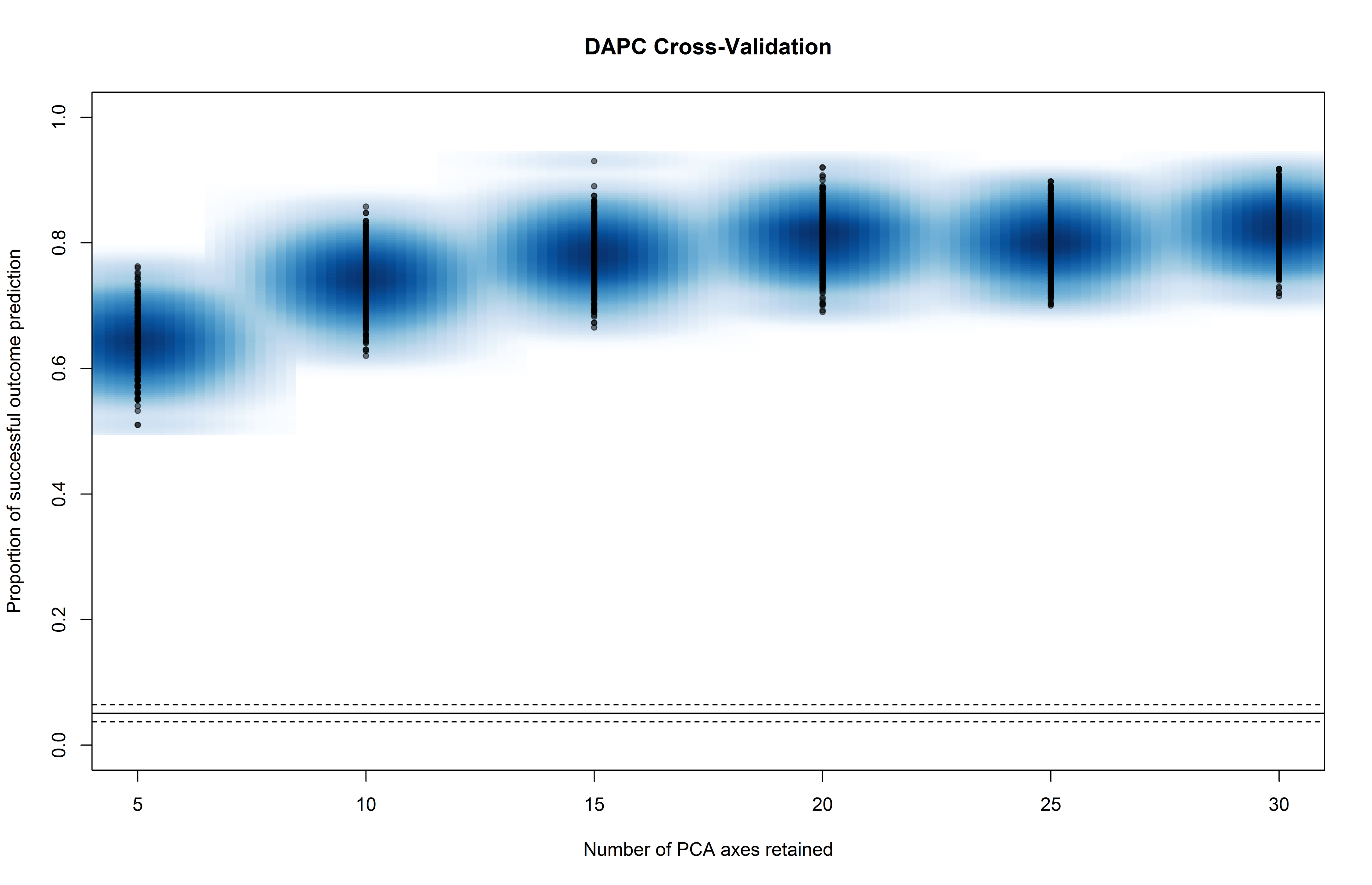

Supplement: S3 Fig — x axis reports the number of PC retained in each DAPC and y axis the proportion of successful outcome prediction. (TIF) [file pone.0207475.s006.tif]

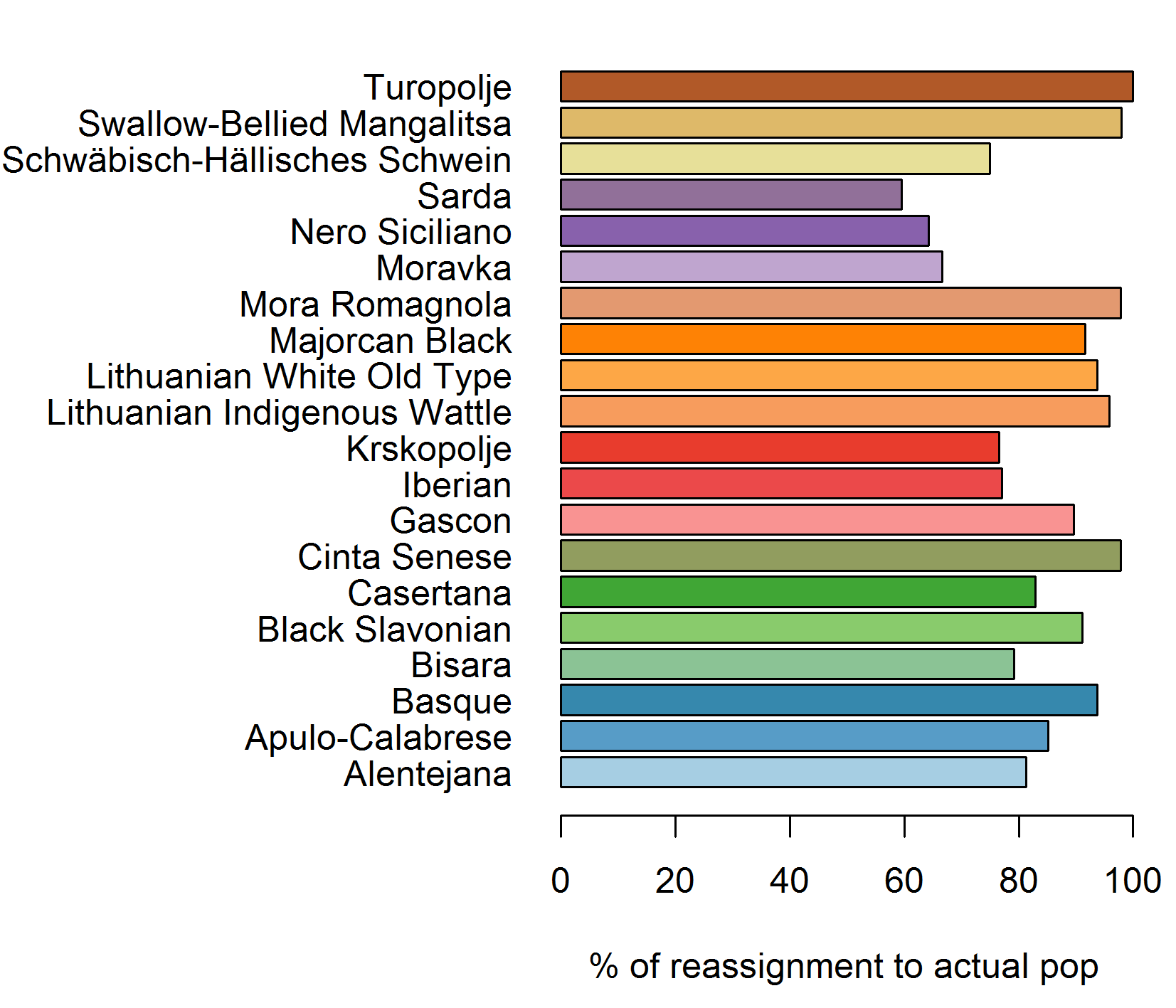

Supplement: S4 Fig — (TIFF) [file pone.0207475.s007.tiff]

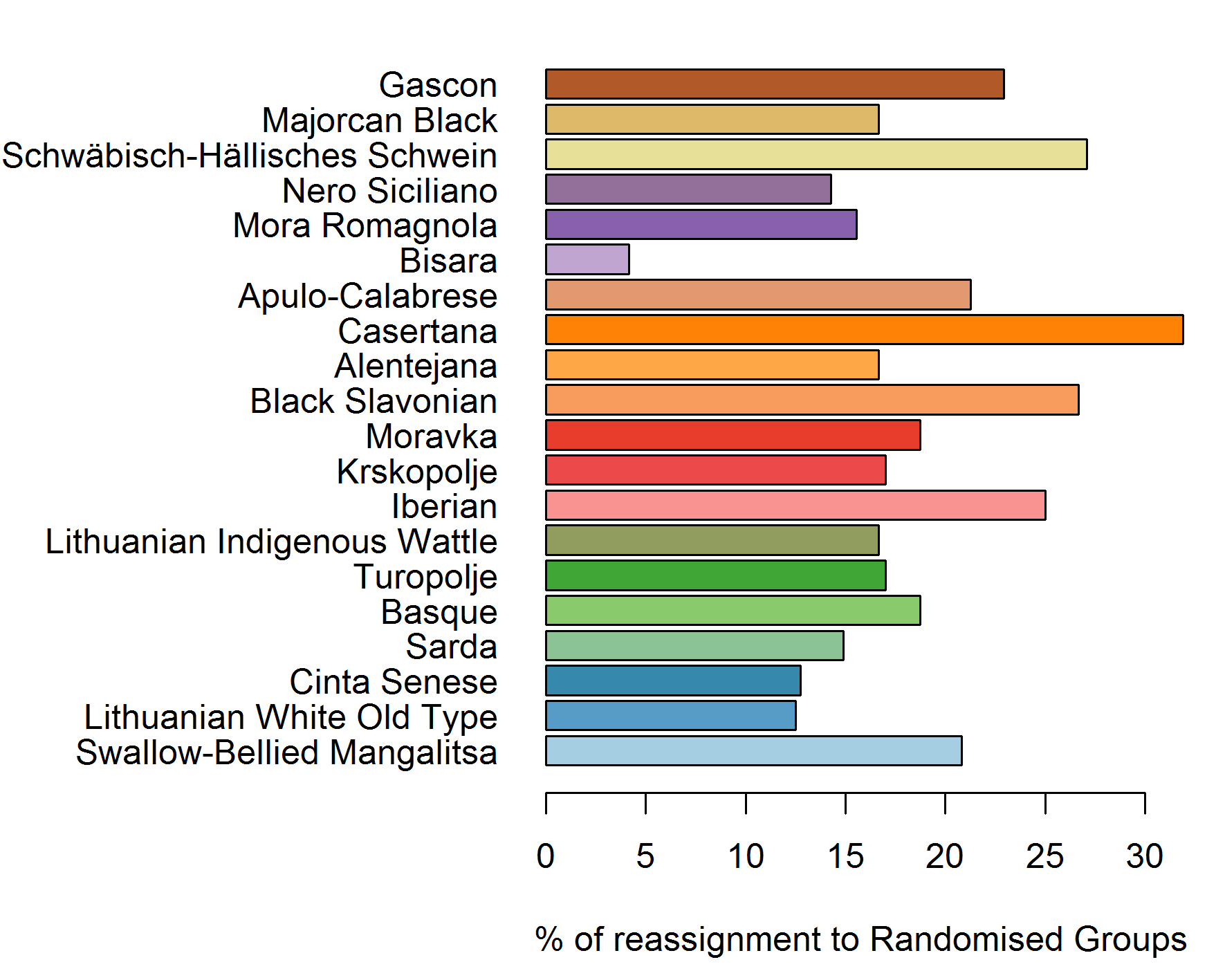

Supplement: S5 Fig — (TIFF) [file pone.0207475.s008.tiff]

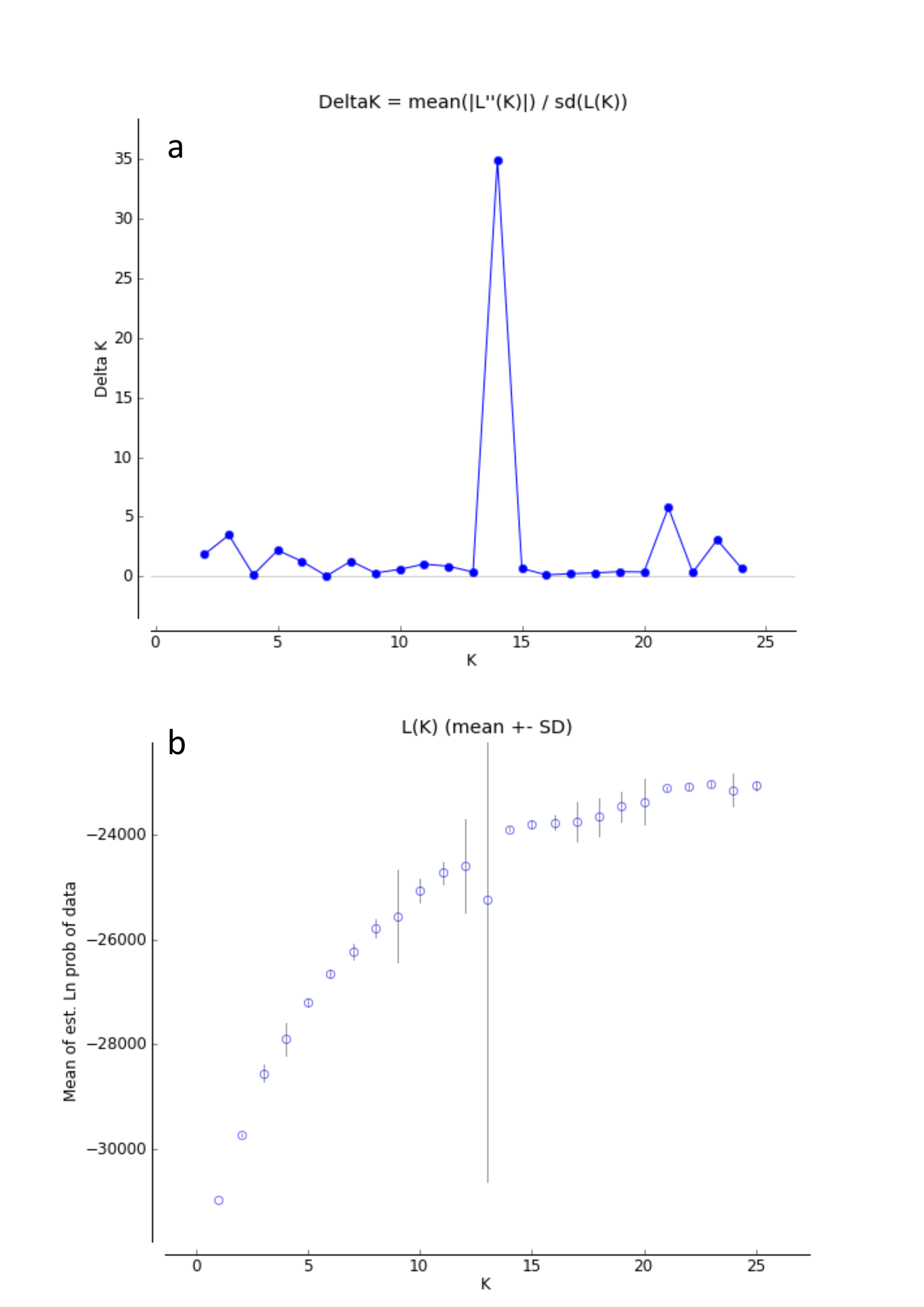

Supplement: S6 Fig — a) plot of ln Pr(G|K) values as a function of the number of clusters for each of the 20 runs carried out for each K value using the program STRUCTURE. b) plot of K as the mean of the absolute values of L”(K) averaged over 20 runs divided by the standard deviation of L(K) between successive K values. (TIF) [file pone.0207475.s009.tif]
